# Aortic dissection as a disease of vascular wall homeostasis: integrating vasa vasorum-inflammation-metabolism axis for mechanistic insight and clinical translation

**DOI:** 10.3389/fimmu.2026.1878406

**Published:** 2026-08-06

**Authors:** Ruilin He, Fengmei Zhang, Haiyan Chen, Jiawei Guo

**Affiliations:** 1 Department of Vascular and Endovascular Surgery, The First Affiliated Hospital of Yangtze University, Jingzhou, Hubei, China; 2 Department of Pharmacology, School of Medicine, Yangtze University, Jingzhou, Hubei, China

**Keywords:** aortic dissection, metabolic–inflammatory coupling, NLRP3 inflammasome, vasa vasorum, vascular homeostasis

## Abstract

Aortic dissection (AD) has traditionally been viewed as an acute structural failure initiated by an intimal tear. However, accumulating evidence suggests that this catastrophic event represents the endpoint of a prolonged, multiscale process of vascular wall homeostatic imbalance. In this review, we propose an integrative framework in which AD arises from the progressive destabilization of a tightly coupled system involving endothelial function, vascular smooth muscle cell phenotype, extracellular matrix integrity, and vasa vasorum dynamics. Central to this model is the vascular–inflammation–metabolism axis, where microvascular dysfunction induces hypoxia, metabolic reprogramming, and oxidative stress, thereby triggering inflammatory amplification and matrix degradation. These processes interact through nonlinear feedback loops, gradually reducing the mechanical resilience of the aortic wall and driving it toward a critical transition. Importantly, the intimal tear is redefined not as the primary cause but as the final manifestation of underlying biological instability. This systems-level perspective reconciles clinical heterogeneity, including variable susceptibility, diverse disease trajectories, and cases lacking clear primary tears. By linking molecular, cellular, and biomechanical mechanisms into a unified framework, this model provides a foundation for identifying early biomarkers, refining risk stratification, and developing targeted therapeutic strategies aimed at restoring vascular homeostasis before catastrophic failure occurs.

## 1 Introduction

In traditional pathological descriptions, aortic dissection has long been regarded as a serious pathological event characterized by the sudden failure of the structural integrity of the vessel wall. The classic pathogenetic process consists of a series of changes in which high-pressure blood in the lumen penetrates the media through a primary tear in the intima, spreads between the layers, and forms a false lumen (pseudolumen). This process further exacerbates the delamination of the vessel wall and the compression of the true lumen (1–3). Morphologically, the triad of coexistence of true and false lumen, intimal flap, and fragmentation of elastic fibers in the media is observed, which is widely recognized as characteristic pathological findings of aortic dissection (4). Historically, the disease was therefore mainly regarded as a structural failure based on mechanical instability; that is, the structural damage occurs as a direct trigger when the vessel wall can no longer withstand the hemodynamic stresses (5).

This idea has influenced clinical practice. Recent advances in early imaging focused on evaluating structural lesions in detail. In high resolution computed tomography angiography and magnetic resonance imaging, the true and false lumen shape, intimal tear location, and the hemodynamic changes can now be accurately described (6, 7). Similarly, treatment followed an approach focused on healing vascular tissue and preserving luminal integrity. Whether surgeons were replacing diseased aorta or stent-grafting to close the intimal tearing, the basic idea was to keep the blood flow into the intima-media domain. In such an approach, structural failure has been treated for the starting point of the disease and its pathological properties (8). However, as observational methods are further advanced in the microscopic realm, this “intimal tear-based model” become less and less convincing. In both high-resolution imaging and pathohistological results, a number of notable changes of the vessel wall are seen. These include a smaller number of smooth muscle cells in the media, fragmentation and disorganisation of elastic fibers, and focal concentrations of mucoid extracellular matrix components (9, 10). Such changes are likely not due to the cause of acute structural failure, but simply due to a long-term remodeling. Additionally, in the affected areas, dilatations and structural anomalies are often observed and their distribution is largely close to those of media losses. This suggests that change is present in the medial extracellular matrix of the the aortic wall in the vessel before it is broken (11–13). That is, the sudden structural failure is preceded by a disease phase that could be termed a “pre-dissection instability phase” and barely detectable, which is by conventional macroscopic imaging.

Molecular evidence also supports this argument. Multi-omics studies show that in the dissected aortic tissue the tissue not only remodels passive proteins but also significantly remodels the cell functions. Vascular smooth muscle cells drop the contractile phenotype, vital for blood circulation, and become a synthetic or inflammation-related phenotype. These cell changes include reprogramming of the extracellular matrix synthesis and degradation pathways, increased expression of inflammatory mediators, and cell migration (14, 15). Matrix metalloproteinases increase, thereby degrading elastic fibers and collagen, and gradually weakening the strongly ordered supporting structure of media. Inflammatory cell infiltrates and oxidative stress also occur in the damaged regions, forming a long-term tissue-remodeling environment (16, 17).

Given this background, one may want to understand aortic decomposition as a result of long-term accumulation of dysregulations of biological homeostasis in some mechanical conditions to critical decomposition. As the balance between cell phenotype, matrix homeostasiness, and local microenvironment becomes weak, the mechanical robustness of the wall may gradually weaken without forewarning (18). The intimal tear or blood entry in the media happening at this time may then be viewed as the origin of the disease, but instead as the final event that brings an underlying subcritical instability state into a sharp structural failure. Such a concept refines the aortic dissection from a vascular-biological point of view, rather than from a purely structural lesion. The wall is not a passive structure that is subjected to hemodynamic load but rather, a dynamic system in which smooth muscle cells, extracellular matrix, microvascular networks, and the local immune response are coupled. The breakdown of homeostasis among these interacting layers ultimately leads to macroscopic structural failure that has a prior biological basis.

### 1.1 Literature search strategy and synthesis approach

We conducted systematic searches in PubMed, Web of Science, and Scopus from inception through May 2026 using combinations of MeSH terms and keywords that covered five themes: aortic dissection per se; inflammation and immunity; vascular smooth muscle cell (VSMC) phenotype and endothelial function; extracellular matrix (ECM) remodeling; and vasa vasorum dynamics with Boolean operators (“OR” for synonyms, “AND” for intersection between domains). We also manually searched reference lists of major guidelines (especially the 2022 ACC/AHA guideline), as well as key reviews, to identify seminal studies not yet indexed.

We included original research (basic, translational, and clinical) as well as systematic reviews, meta-analyses, and official guidelines that were published in English. Case reports with fewer than three cases; conference abstracts; editorials without original data; and studies limited only to peripheral arterial dissections not relevant to the aorta were excluded from this review.

**Figure 1:**
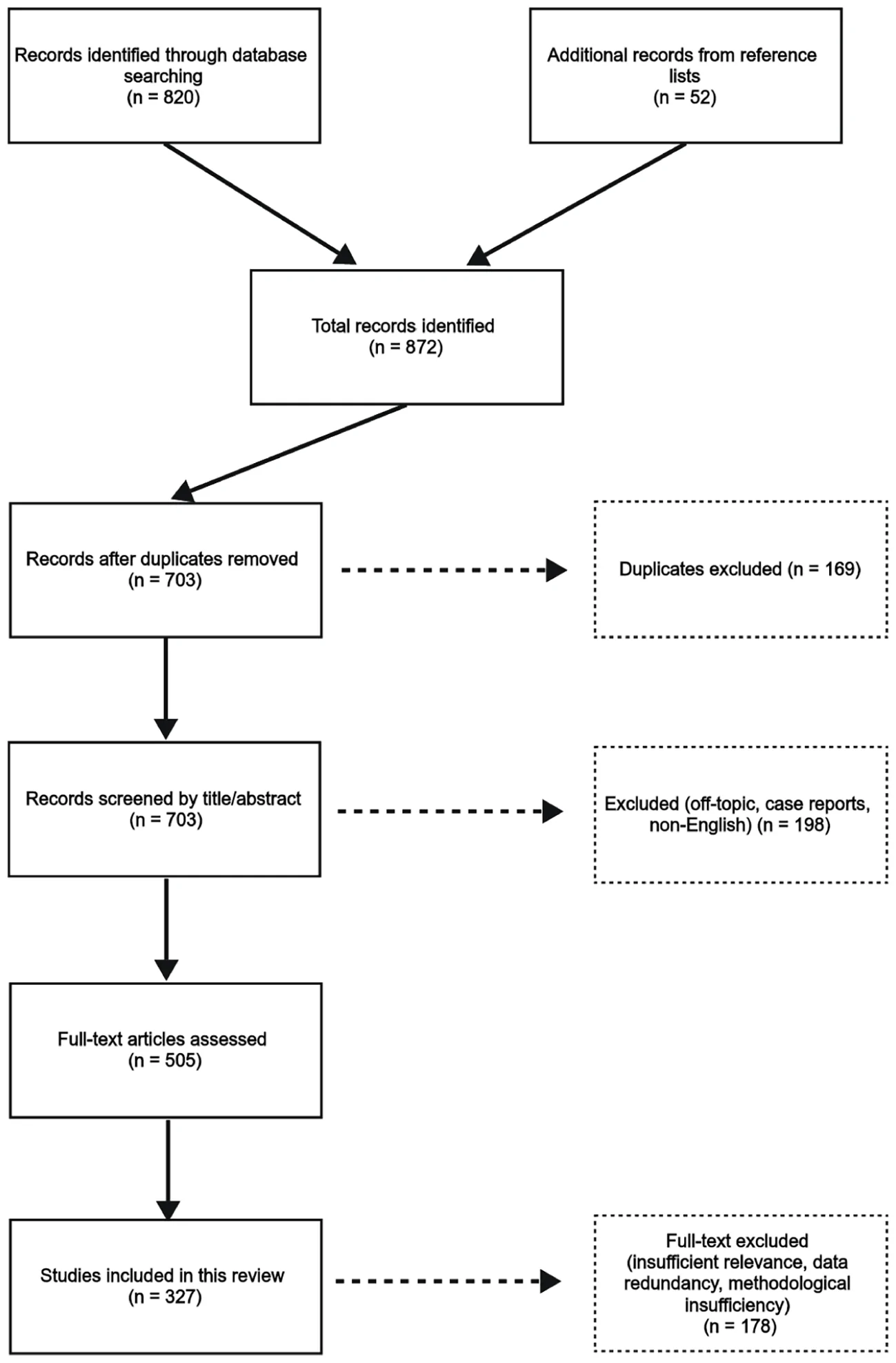
Flow diagram of the systematic literature search and article selection.

The initial search yielded 872 records. After duplicate removal, 703 unique records remained. Title/abstract screening excluded 198 off-topic or ineligible items, leaving 505 full-text articles for detailed assessment. Of these, 178 were further excluded due to insufficient relevance to our five core subsystems, data redundancy, or methodological insufficiency. Ultimately, 327 articleswere included as the citation foundation of this review. A PRISMA-style flow diagram detailing each screening step is presented in **Figure 1**.

Because of the large variation in the types of evidence we have collected—from single-cell/spatial transcriptomics and Mendelian randomization through computational biomechanics and clinical registries—we conducted an integrative narrative synthesis based on theories instead of a quantitative meta-analysis. We identified key findings that could be mapped into the five predetermined interacting subsystems (inflammation, endothelium, VSMC, ECM, and vasa vasorum) focusing on their bidirectional crosstalk, feedback loops, and convergent mechanistic nodes (mechanotransduction, metabolic reprogramming, and inflammasome activation). In this way, we developed a coherent dynamic model for progressive aortic wall destabilization, which reframed the intimal tear as a late stage of protracted preclinical wall deterioration.

### 1.2 The central contradiction confronting the traditional structural model

One of the common issues in clinical practice is that only an intimal tear caused by blood can cause aortic dissection, which implies that imaging and pathology results are generally homogeneous. On more and more occasions, the etiology is far more difficult. First, there are far more cases where no distinct primary intimal tearing is identified. Imaging and pathohistological studies have consistently confirmed a disease course wherein a local hemorrhage at the intima-media boundary leads to an evolving intramural hematoma, which then communicates with the true lumen and forms a typical aortic dissection (19, 20). This indicates that in some cases, the microvascular system of the vessel wall (Vasa vasorum) may be damaged or more permeable at first, therefore blood is not necessarily bound to the wall from the luminal side but may, in some situations, originate from the wall’s microvessels.

Another important aspect of clinical development that cannot be explained from a purely mechanical perspective is the variability of susceptibility between people: the aorta is subjected to strong blood pressure, shear stress, and local stress at bends. However, even in individuals with similar blood pressure and hemodynamic conditions only a small fraction of them complete their interior and most are able to maintain the structure for a long time, but severe structural failure of the wall of vessels is observed with no major variation of blood pressure (21, 22). This asymmetry appears hard to explain from a pure mechanical viewpoint, and suggests that the inherent vulnerability of the vessel wall - i. e. functional state of the smooth muscle cells, metabolic capacity of the extracellular matrix, and the local inflammatory environment –is responsible for this differential susceptibility. Mechanical stress acts more as a trigger than as the root cause (23).

The remarkable heterogeneity of the disease course also limits the explanation for the purely structural model. The patients have very different expectations about the speed of spread of the dissection, the stability of the false lumen and the long-term risk of aneurysmal dilatation. Many individuals remain relatively stable after the acute phase, while others undergo gradually increasing false lumen enlargement or repeat dissections (24–26). The traditional classification using anatomical location (such as Stanford type A and type B) is helpful for acute care, but its predictive value for long-term vascular remodeling or recurrence risk is not reliable. These uncertainties show that aortic dissection is not a self-contained process but a dynamic process driven by the role of vascular cell behaviour, matrix metabolism and local microenvironmental changes (27–29).

In conclusion, the hypothesis that a segment is simply due to an “intimal tear from hemodynamic stress” no longer makes sense for the current evidence of clinical and experimental interest. The different trajectories of intramural hematoma, differences in individual susceptibility, and pronounced heterogeneity of this disease course are all consistent. Collectively, these observations support that dissection is a critical event due to a progressive degradation of vessel wall homeostasis and the intimal tear is the final macroscopic manifestation of the process. This new understanding gives us the theory needed to investigate the underlying biological processes of the vessel wall (microvascular system, smooth muscle cell state, remodeling of extracellular matrix) as mechanisms that control the development and progression of the disease.

### 1.3 “Multilayer homeostasis imbalance” – an integrative framework for the pathology of aortic dissection

In classical mechanics, aortic dissection was thought of as a sudden structural collapse. The hemodynamic impulse tears the intima, blood flows through the media, spreads through the layers and forms true and false lumen anatomical structures (30–32). While this interpretation is supported by macroscopic and morphological evidence, its impact decreases with time from the structural to the cellular level. The vessel wall is not a passive tissue merely subjected to blood flow, but a continuously and tightly regulated biological system (33). The endothelial barrier protects homeostasis at the blood– vessel wall interface; vascular smooth muscle cells regulate contractile function and matrix metabolism through phenotype switching; the extracellular matrix is not only a scaffold with mechanical loads, but also transmits mechanical signals through integrins and cytoskeleton (34, 35). Further complicating this system are tissue-resitu immune cells and cytokines, metabolic intermediates, and chemotactic signals which continuously regulate the functional state of the various components. Oxygen gradients, mechanosensitive signals, and metabolic fluxes are closely linked and are found to form a very closely interwoven homeostatic network between large spatial scales (36).

From this perspective, aortic failure could be seen more precisely as a direct consequence of a progressive balancing of this multilayer regulatory system inside the vessel wall. It does not cause a failure of a single component, but rather is a slow destabilization process. Endothelial barrier failure, phenotype switching of smooth muscle cells, and anomalies in the metabolism of the extra-cellular matrix both produce small changes in the mechanical adaptivity of the vessel. When microvascular remodeling and inflammatory signaling are added locally, local oxygen supply and metabolic state are different (37). The feedback loops that maintain interlaminar balance are asymmetrically amplified, and the vessel wall progressively loses with its mechanical tolerance. In this case, the apparent event of an incidental intimal tear or bleeding within the media may in fact be the event that transitions an unstable state into collapse (38).

This view of multilayer homeostasis imbalance can be used to solve some important clinical problems. For example, this fact that some of the cases are intramural hematoma means that functional disruption of the microvascular network of vessel wall can occur late in the disease. A rupture or fracture of the vasa vasorum can cause local bleeding sites in media and local mechanical conditions. Structural or molecular anomalies due to genetic background (mutations in matrix protein assembly or intracellular signaling pathways in smooth muscle cells) can reduce the time available for the vessel wall to adopt to hemodynamic stress (39, 40). When this vulnerability and environmental conditions (hypertension, diabetes, etc.) play a role, these factors can systematically cause cell metabolic regimes and mechanosensitive pathways to display local structural destabilization.

Once these events do not stop, inflammatory process and metabolism become passive effects and significant amplifications of disease. Inflammatory cells, oxidative stress, and continuous activation of matrix degradation pathways not only alter the structure of the extracellular matrix, but also break the phenotype of smooth muscle cells which drives vessel wall into a self-amplifying remodeling process. Matrix degradation leads to cell migration and inflammatory signals; the inflammation microenvironment carries up matrix turnover. In this process, the internal robustness of the vessel wall decreases, and the later hemodynamic fluctuations are more likely to become fatal.

Overall, aortic damage may not just be considered as a structural event only in time but rather as a dynamic pathological process through molecular, cellular, and tissue levels. The interactions between levels connect seemingly unrelated pathological phenomena and bring disparate observations into a coherent logic. This systematic perspective gives a new theoretical model of when the disease onset, each of its susceptibility, and different courses take place, and point to specific targeting directions for future translational research. If vessel wall homeostasis breaks before it happens, important opportunities for clinical intervention may be at this point.

**Figure 2:**
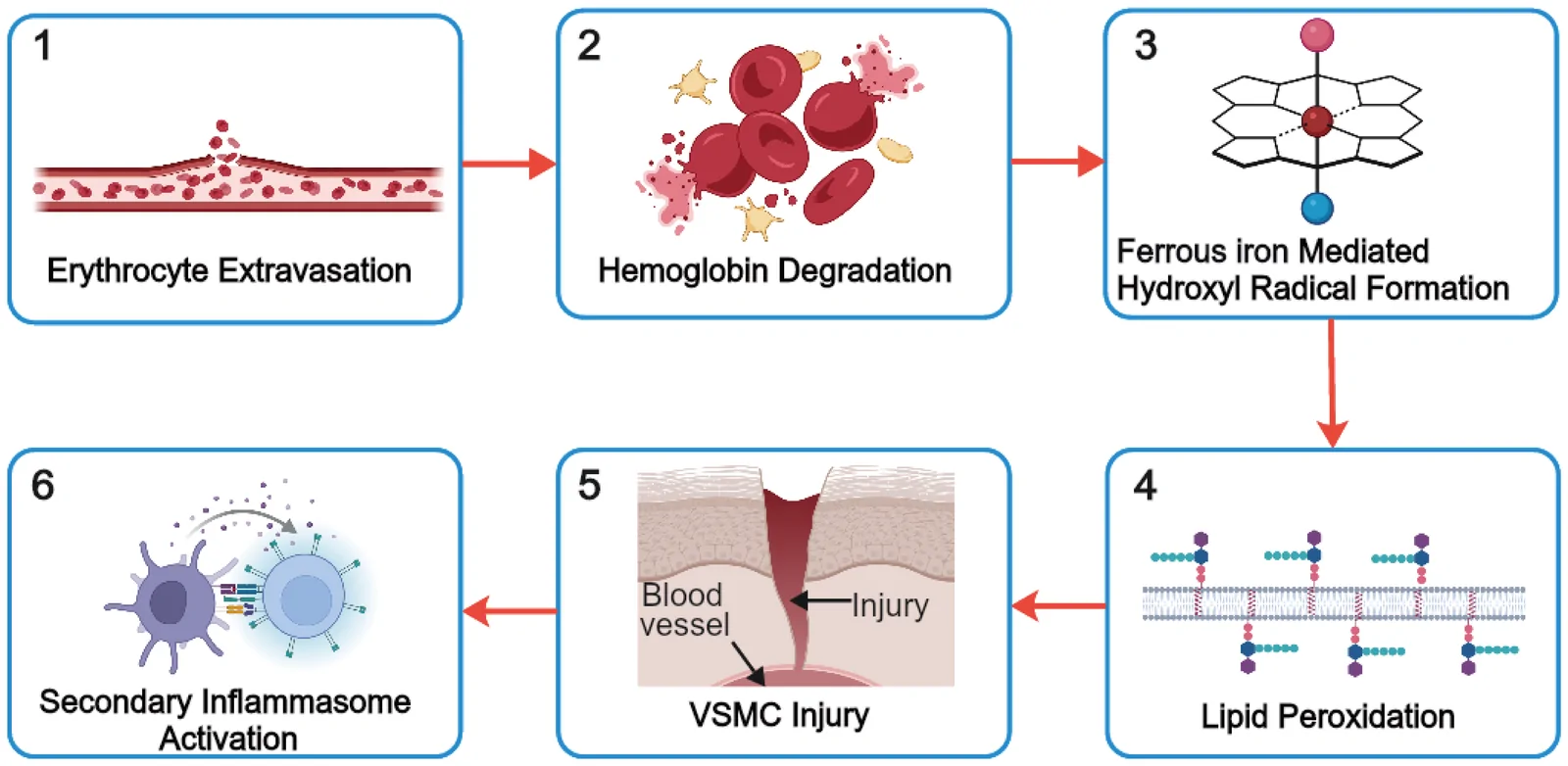
Iron-driven oxidative–inflammatory cascade following vasa vasorum microhemorrhage.

**Figure 2** presents this iron-driven cascade reaction, originating from microhemorrhage of the outer membrane’s nourishing vessels, in a schematic diagram. As shown in the figure, when the fragile pathological new blood vessels rupture, red blood cells infiltrate the interstitium of the vessel wall. Subsequently, the release of hemoglobin leads to the release of free iron ions, which, through the Fenton reaction, catalyze the production of a large number of hydroxyl radicals, triggering intense oxidative stress. This process not only directly damages the surrounding vascular smooth muscle cells, promoting their transformation into iron death and other pro-inflammatory death modes, but more importantly, the oxidative stress products, as endogenous danger signals, will activate the local innate immune response, thereby amplifying an isolated micro-hemorrhage event into a persistent oxidative-inflammatory vicious cycle. This provides a crucial mechanistic connection point for understanding the “from outside to inside” pathogenesis hypothesis of aortic dissection.

## 2 Redefinition of the disease concept: imbalance of vessel wall homeostasis

### 2.1 Systematic definition and dynamic characteristics of vascular homeostasis

Biological stability of the aorta cannot be viewed as a static link between independent structural or molecular subsystems. Instead, the a-wall serves as a controlled and multiscale biological system in which mechanical, metabolic and inflammatory signals are coupled with the re-organization of the extracellular matrix at across spatial and temporal scales. The structural integrity of the vessel thus depends not only on the stability of individual components, but also on the coordinated regulation and adaptation of multiple subsystems (41, 42).

At the microscopic scale, the pulsatile blood flow periodic strains and shear stresses influence vessel shape and structure at the microscopic level. Endothelial cells and vascular smooth muscle cells transmits mechanical signals to the cell via mechanosensitive pathways which regulate vascular tone, barrier permeability, and cell shape. In the opposite direction, intracellular networks maintain energy supply and redox balance, and the extracellular matrix undergoes constant remodeling characterized by matrix metalloproteinases and endogenous inhibitors. These two processes form a hierarchical regulatory network that maintains the structural and functional stability of the vessel wall under the physiological conditions (43, 44).

From a systems biology point of view, the aortic wall is a dissipative structure in non-equilibrium thermodynamics. Through continuous energy input of blood flow, the system remains out of equilibrium but remains highly ordered and robust (45). Such dynamic stability requires efficient signal transmission between the intima, media and adventitia. Mechanical signal disturbances at the luminal side are detected within milliseconds by mechanoreceptors on the endothelial cells - ion channels, integrins and glycocalyx - initiating early signal responses, amplifying over minutes to hours through transcriptional and epigenetic reprogramming, and resulting in a changing functional state of endothelial and smooth muscle cells (46, 47).

In physiological conditions such a multilayer network has a remarkable capability to absorb and adapt to the transient events. For example, lower laminar shear stress can be compensated by increased nitric oxide production and release of vasodilatory signals. Local damage to the extracellular matrix triggers reparative matrix formation, and transient inflammatory activation typically is limited by efficient anti-inflammatory and pro-resolving mechanisms. These negative feedbacks are the major source of vascular homeostasis (48–50).

However, when multiple axis of regulation is also blocked, the system becomes overwhelmed. Endothelial erosion-induced barrier defects, mitochondrial failure, oxidative imbalance and other metabolic stresses are accompanied by faster extracellular matrix degradation, which results in vessel wall collapse (51, 52). The regulated processes that normally slow fluctuations may turn into self-amplifying positive feedbacks. Inflammatory signals regulate expression and activity of matrix metalloproteinases, increase extracellular matrix degradations, and damage-associated molecular patterns (DAMPs) released from the degradated matrix also increase immune activation, thus maintaining inflammation. Interactions in other ways, including phenotype switching from contractile to synthetic or pro-inflammatory phenotypes, further worsen the structural weakening of vessel wall (53–55).

When they start, this pathological feedback can propagate along the vessels wall, disrupt the communication between the layers, and then reduce structural content. In this scenario, aortic dissection is viewed not as a simple local intimal tear or a single molecular defect, but as a macroscopic collapse of the vascular homeostasis system. This overall perspective sets a global theory that allows us to make connections across mechanical, cellular and molecular mechanisms, giving new opportunities for early disease detection and targeted therapy.

### 2.2 Molecular and cellular evidence for the multilayer steady-state imbalance

#### 2.2.1 Endothelial cell destabilization: collapse of the vascular mechanotransduction interface

Endothelial cells operate at the interface between blood and vessel wall, an active vessel wall with mechanical and biochemical signals. Via a highly specialized mechanotransduction system, they turn the hemodynamic forces into coordinated transcription, metabolic, and paracrine response for vascular tone, immune monitoring and communication in the vessel wall.

The process is accompanied by several complementary mechanosensory pathways. In physiological laminar shear stress, a transcription mechanism generated by KLF2 and KLF4 produces anti-inflammatory anti-thrombotic endothelial phenotype, increases nitric oxide production and degrades adhesion molecules (56–58). Meanwhile, mechanical signals transmitted through the cytoskeleton control gene transcription by Hippo pathway effects YAP and TAZ, correlating matrix stiffness and shear shearing to nuclear transcription. On smaller timescales mechanosensitive ion channels such as Piezo1 initiate calcium influx within seconds, triggering intracellular signaling cascades to adjust endothelial response to dynamic blood flow (59–61). In addition, it has been indicated that the endothelial glycocalyx and integrin-mediated focal adhesions are key elements of the mechanoreceptor complex that moves forces from endothelium into intracellular signaling (62).

In the context of aortic disease this highly integrated mechanotransduction network deteriorates early on. In diseased aorta tissue, KLF2 and KLF4 expression drops, endothelial cells die and pro-inflammatory transcription programs are activated (63, 64). As an additional effect, impaired endothelial nitric oxide synthase production reduces nitric oxides and the central vascular protection functions – platelet aggregation, leukocyte adhesion, and vasodilation. This decreased nitric oxide bioavailability also promotes endothelial cell activation, showing upregulation of adhesion molecules like VCAM-1 and ICAM- 1, which facilitates leukocytes recruitment and adhesion (65, 66).

Beyond transcription, the breakdown of endothelial junctional structure is key to the cell-cell vascular adhesion in homeostasis. Cell-cell binding within the endothelial monolayer is maintained by VE-cadherin-centered adherens connections (67). Inflammatory cytokines, oxidative stress, and abnormal shear stress degrade VE-cadherin. This contributes to higher endothelial permeability, and diffusion of plasma proteins, circulating cytokines and immune cells in the subendothelial space and media layers (68). This permeability is not only localized but also reflects a widespread breakdown of the barrier function that is at the core of interlaminar control in the vessel wall. In normal circumstances, the endothelium acts as a selective regulator. The medial smooth muscle cells are protected from pro-inflammatory and pro-thrombotic stimuli from the circulation. When barrier integrity is broken, the biochemical microenvironment of the media changes dramatically, producing inflammatory response, oxidative load and smooth muscle cell phenotype switch (69, 70).

Further studies show that endothelial dysfunction is accompanied by metabolic reprogramming, a glycolytic response and stress in the mitochondria, that amplifies redox imbalance and inflammatory signals (71, 72). The metabolic imbalance adds an additional stress to the vessel wall, connecting energy metabolism damage to structural instability. Overall, mechanotransduction breakdown, lack of nitric oxide signaling, loss of junctional integrity, and metabolic repregulation, all translate endothelial cells from a healthy regulatory interface to a source of pro-inflammatory and pro-remodeling signals. In multilayer homeostasis imbalance, the unstable state of endothelial cell triggers downstream intramural damage and eventually aortic dissection.

#### 2.2.2 Phenotypic reprogramming of vascular smooth muscle cells

In the aortic medium, vascular smooth muscle cells are the load-bearing cell and must maintain mechanical integrity. In physiological environments, they exhibit a highly specialized contractile phenotype and highly express contractile marker proteins, such as α-smooth muscle actin (α-SMA) and SM22α that coordinate to generate tension and give a uniform distribution of circumferential wall stress in the course of the heart cycle (73–75).

Another trait of vascular smooth muscle cells is their phenotypic plasticity. When exposed to environmental perturbation, inflammatory cytokines, oxidative stress, abnormal TGF-β signaling, and extracellular matrix degradation, the cells switch from a contractile to a synthetic, inflammatory, and even osteogenic phenotype, downregulating contractile markers and upregulating genes involved in remodeling the matrix, cytokine secretion and immune modulation (76–78).

This phenotype is controlled by regulatory networks. Dysregulation of the TGF-β/SMAD signaling pathway limits smooth muscle cell differentiation and matrix homeostasis in hereditary aortic disease such as Marfan syndrome or Loeys-Dietz syndrome. Activation of inflammatory pathways such as NF-κB promotes pro-inflammatory transcription and oxidative stress enhances its effects via oxidation-sensitive signalling channels (79, 80). Recent studies also indicated that epigenetic processes such as chromatin remodeling and non-coding RNAs also regulate the pathological smooth muscle phenotype (81).

Recent advances in single-cell RNA sequencing reveal the heterogeneity of smooth muscle cell in diseased aortas at the microscopic level. In this study, several subpopulations with mixed transcriptional states, co-expressing genes corresponding to contractile function, inflammatory response and stress signaling, and a subpopulation with high levels of senescence, apoptotic signaling and pro-inflammatory mediators may participate in the pathological microenvironment, suggesting that it is a phenotypic diversification and rather than a uniform degenerative process (82, 83).

Apart from phenotype switching, changes in the absolute number of smooth muscle cells—particularly loss of contractile cells—compromise vessel wall integrity. Apoptosis, necroptosis, and senescence progressively decrease the number of active contractile cells. As the number of competent load-bearing cells decreases, the mechanical stress gets more unevenly distributed around the extracellular matrix scaffold, and the vessel wall slowly becomes a cell-rich landscape with cell-poor regions and structural discontinuities (84).

These microstructural defects have much lower limits of mechanical failure. Regions with smaller numbers and impaired smooth muscle cells become stress concentrations in the aortic wall, which can become easily subjected to intima-media delamination under pulsatile conditions. Here, the false lumen in aort surgery can be viewed as the macroscopic expression of cumulative cellular dysfunction and mechanical destabilization.

#### 2.2.3 Imbalance of the extracellular matrix: from adaptive remodeling to structural vulnerability

The mechanical stability and quality of the aorta ultimately comes from a highly ordered extracellular matrix composed primarily of joint layers of elastic fibers and collagen fibers. The two give complementary biomechanical properties: the elastin is elastic, reversible deformation occurs under cyclic loading and the collagen fibers are tensile, constrained by excessive growth at high pressures (85). Together, they allow the aorta to serve as an energy buffer in cardiovascular system.

The extracellular matrix is a passive structural scaffold but a bioactive system that is continuously remodeling through mechanical and biochemical signals. Smooth muscle cells act as the regulators of matrix metabolism due to blood flow and environmental signals while simultaneously being highly regulated in terms of matrix synthesis and degradation (86, 87).

In the diseased case this balance is violated, and structural integrity degenerate. One common complication of aortic tissue is that matrix metalloproteinases (MMP-2 and MMP-9) are up-regulated, and insufficient expression of TIMPs. This proteolytic imbalance leads to the degradation of elastic fibers, the elastic recoil capacity which buffers pulsatile loads, and the susceptibility to mechanical fatigue (88).

Degraded elastin fragments, also known as elastin-derived peptides (EDPs), act as damage-associated molecular patterns (DAMPs) via binding to pattern recognition receptors in immune and vascular cells to activate pro-inflammatory signalling pathways such as NF-κB. A vicious cycle of these process is the growth of matrix degradation and inflammation increases protease activity, which causes tissue damage (89, 90).

Along with this, collagen remodeling modifications also distort the aortic wall’s mechanical structure. Degraded collagen fibers, reduced cross-linking stability, and fiber network disruption will cause uneven distribution of tensile strength. Impaired cross-linking (e.g., due to lysyl oxidase dysfunction) and disordered collagen deposition degrade the matrix quality and the extracellular matrix loses the functional network information required for coordinated load transfer (91).

From a biomechanical point of view, these changes indicate a change from a load-bearing system to a system of stress concentrations. In healthy aorta, mechanical forces occur on the highly interconnected matrix network, while in diseased conditions matrix decay and disorganisation lead to local stress amplification (92, 93). Once the region is sufficiently close to a critical point they become mechanical weak points, and may be susceptible to intimal tearing and propagation along paths of least resistance.

Extracellular matrix remodeling is associated with cell function and mechanosensitive signaling pathways. Changes in matrix stiffness and composition increase smooth muscle cell phenotype via integrin signaling, cytoskeletal reorganization, mechanosensitivity and the cycle of inflammation, metabolic stress, and structural damage (94). This direction of interaction suggests that the matrix is not only the main cause of disease but also one of the major drivers of pathology.

In the multilayer homeostasis condition, extracellular matrix degradation is not only a consequence of endothelial dysfunction and smooth muscle cell phenotype switching, but also an active modulation of pathological signals. The combination of proteolysis, inflammation, metabolic stress and mechanical destabilization establishes a structural basis for aortic remodeling.

#### 2.2.4 Dysfunction of the vasa vasorum: destabilization signals originating from the adventitia

The outer and inner layers of the aortic wall are fed oxygen and nutrients by a microvascular network called vasa vasorum. In normal conditions such a system may also respond to the needs of the adventitia and outer media but pathohistological analysis of diseased aortics often shows remodeling (95).

Another common scenario is angiogenesis (new blood vessel formation) of the aortic surface, even before an overt aortic dissection occurs (96). Vascular endothelial growth factor (VEGF) helps to establish new vessels, but they are often immature, possessing only a partial basement membrane and weak endothelial junctions, leading to a greater permeability and leaky vessel (97, 98).

If such fragile vessels rupture or leak, erythrocytes invade the surrounding tissue and hemolysis releases hemoglobin and iron metabolites due to oxidative reactions. Local accumulation of these substances causes oxidative stress and iron-mediated oxidative damage. Microhemorrhaging events also generate local hypoxic microenvironments in the media (99).

Hypoxia, in turn, activates HIF-1α transcription programs that promotes inflammation and matrix remodeling. The role of hypoxia and oxidative stress and inflammatory activity suggests that changes in the vasa vasorum may be an early source of pathological damage, not simply a side effect of vascular injury (100, 101). Such processes, which originate in the earlyitia, can spread out through media, amplify systemic breakdown of vascular homeostasis and ultimately create aortic death.

**Figure 3:**
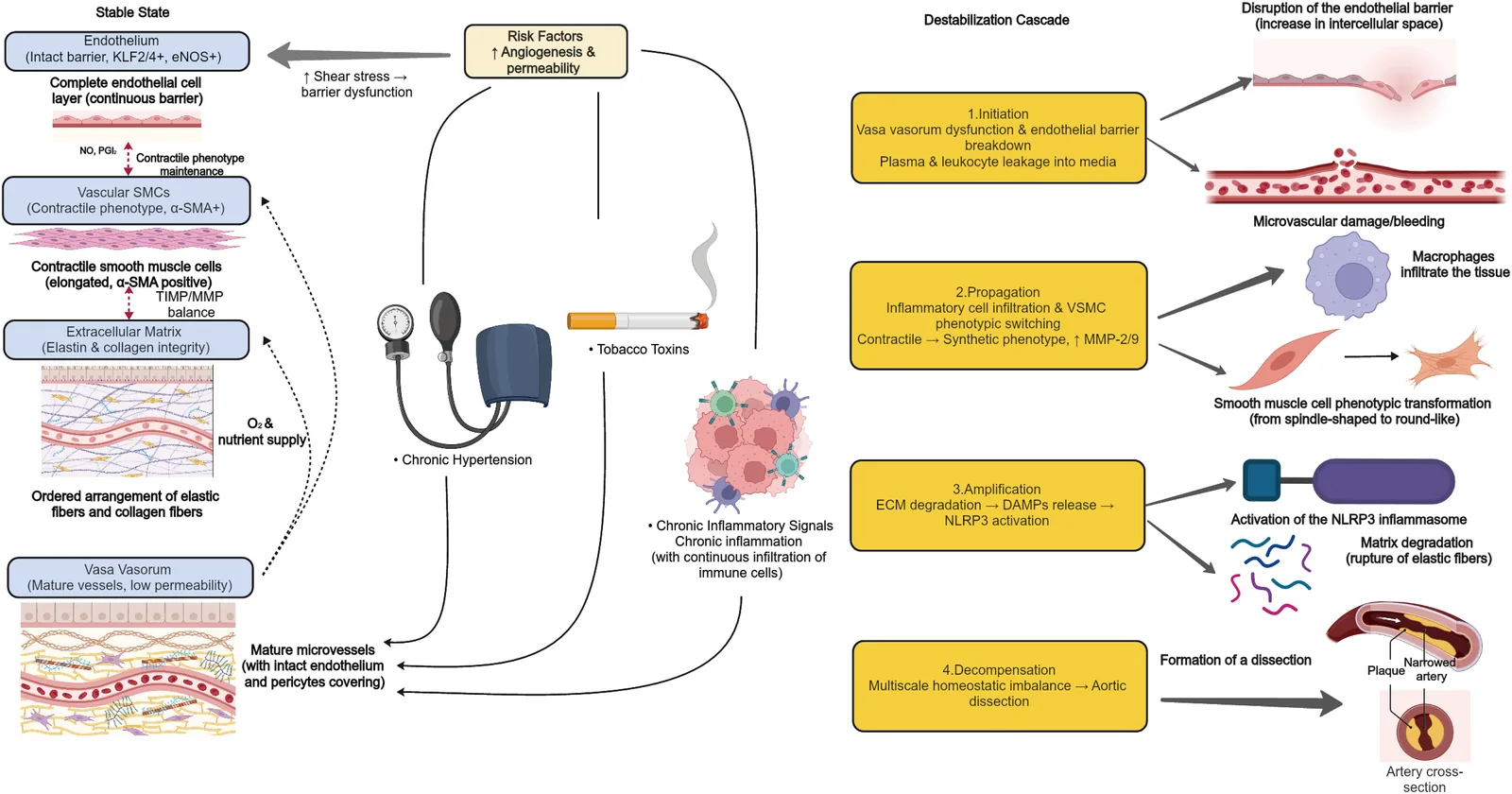
Multiscale homeostatic imbalance in aortic wall leading to dissection.

**Figure 3** provides an intuitive illustration of this concept. It depicts the aortic wall as a dynamic functional network composed of the inner layer, the smooth muscle middle layer, the external membrane nutrient vessels, and the extracellular matrix. Under a stable state, the various layers maintain a balance of mechanical and biological functions through precise signal interactions. However, when various risk factors cause dysfunction in one or more links, a cross-level cascade reaction will occur, such as the disruption of the endothelial barrier leading to the infiltration of inflammatory cells, the exacerbation of matrix degradation due to the transformation of smooth muscle cell phenotype, and the formation of pathological new blood vessels as the source of inflammation and bleeding. This multi-scale imbalance of the stable state ultimately jointly drives the development of the vascular wall towards decompensation and dissection.

To present this concept of multi-layered steady-state imbalance more systematically, **Table 1** summarizes the core functions of each key structural layer of the aortic wall – from the endothelial cells in the inner membrane to the nutrient vessels in the outer membrane – in maintaining homeostasis, the evidence of their dysregulation in disease states, and the resulting histological consequences. This table aims to provide a clear framework that links the aforementioned microscopic molecular events with their impact on the macroscopic structural integrity of the vascular wall.

**Table 1:** Multilayer structural and functional homeostasis of the aortic wall and their pathological derangements in dissection.

Structural layer	Homeostatic maintenance factor	Evidence of dysregulation	Histological consequences
Endothelial layer	Shear Force Sensing	KLF2 (↓), eNOS (↓), YAP (Dysreg)	Barrier Leakage, Pro-inflammatory Environment
Middle layer (VSMC)	Maintenance of Contractile Phenotype	α-SMA (↓), Pro-inflammatory Cluster (↑)	Loss of Mechanical Tension, Hyperplasia
ECM	Scaffold Proteolytic Equilibrium	MMP-2/9 (↑), TIMP (↓), Elastin Fragmentation (↑)	Loss of Elasticity, Mechanically Weak Zones
Vessel vessels (VV)	Oxygen Supply Equilibrium	VEGF (↑), Micro-hemorrhage (↑)	Hypoxic Microenvironment, Inflammation Initiation

## 3 The vascular-inflammation-metabolism axis: temporal drivers of aortic wall destabilization headings

In the multilayer homeostasis balance of the vessel wall, a question remains unanswered: if the wall is sufficiently compromised for structural reasons, what mechanism drives the transition from a compensated state to intimal rupture? There is emerging evidence that this transition is not mediated by a single trigger, but the result of an unfolding of a set of interconnected biological processes that develop over time (102).

Both gene and molecular studies consistently reveal microvessels to be a source of early pathological causes (103). In some cases, changes in the vasa vasorum before exiting the media, suggesting that structural damage may not result from the intima as previously suggested (104). The substantial time gap between microvascular anomalies and intimal rupture indicates a new discussion on causal links between these illnesses.

Local hypoxia from microcirculatory disruption may lead to a cascade of adaptive responses that, subsequently, drive the overall remodeling of the vessel wall. Hypoxia does not occur consistently; cells usually change their metabolic strategy between mitochondria oxidative phosphorylation and glycolytic ATP (105, 106). This reprogramming is effective for cell survival, but also changes the redox microenvironment and increases the concentration of signaling metabolites. These metabolic changes are related to inflammatory processes. This lowers the threshold of immune activation and leads to persistent overexproduction of cytokines beyond the initial stimulation.

Once metabolic-inflammatory interaction is established, a self-perpetuating process will then proceed. Proteolytic activity will be increased, extracellular matrix components will deteriorate, and mechanical properties of the vessel wall will degrade (107). In this stage, structural damage is no longer a isolated cause of biological stress. In this context, an anatomical aortic segmentation is the external phenotypic manifestation of a process that has slowly evolved through many regulatory layers and reached its end point.

This also provides a clear explanation of clinical heterogeneity of aortic division. Potential downstream risks such as hypertension or systemic inflammation may take different initial paths but converge to common downstream processes (microvascular dysfunction, metabolic stress, inflammation amplification). While these risks are largely different, there is a complementary dynamic between system dynamics which drives convergence to a common pathway.

### 3.1 Pathological remodeling of the vasa vasorum: a new perspective on the origin of aortic wall destabilization

In clinical conditions the vasa vasorum of the aorta is mainly localized in the adventitia and outer media, and oxygen flows from the body into the vessel wall; their morphology is fixed and maintains a robust balance between perfusion demand and structural stability. Intact endothelial junctions, rich pericyte coverage, and low permeability are a controlled channel for microvascular diffusion and therefore blood fragments are not allowed to flow to the vessel Walls (108, 109).

This stable state may be disturbed by chronic stress. Long-term hypertension, exposure to tobacco toxins and ongoing inflammatory signals drive microvascular growth (110). This vascular growth might seem a way of improving tissue perfusion, but the current models lack mature functioning vessels. Microvessels have a discontinuous basement membrane and a relatively few endothelial gaps. These features show that the vessels are in an unstable and functionally suboptimal, state (111).

These structural abnormalities directly manifest functional effects. High microvascular permeability leads to plasma proteins leaking and circulating inflammatory mediators to the surrounding tissue and in some regions, they lead to areas of erythrocyte extravasation (112, 113). Iron released from hemoglobin breakdown catalyzes oxidative reactions in addition to the local biochemical microenvironment. These changes, not just for the outer layer, can also propagate into the media via molecular diffusion and interlayer signaling.

The increased presence of wall hemorrhages in the absence of an overt intimal tear raises some new questions, such as whether aortic dissection might originate not from the intimal side but rather from an internal structural disturbance within the media and are likely to result in mechanical failure (114, 115). The once- nonstandard “outside-in” hypothesis of pathogenesis is now in agreement with existing histological evidence and mechanistic studies.

If this was true, microvascular dysfunction plays an important upstream role in the pathological process. It is not a step back after tissue damage, but it is the main component of the loss of mechanical elasticity in the aortic wall. The intimal tear, long thought to lead to this end-phenotype of enormous structural stress in the vessel wall, may rather become the end-phantom.

### 3.2 Local hypoxia and metabolic reprogramming: changes in the energy metabolism of the aortic wall

Vascular vasorum degeneration leads to focal perfusion deficits and to a hypoxic environment with periodic reoxygenation, where such fluctuations can exert potent biological effects (116). Maintenance of the transcription factor HIF-1α triggers an extensive transcriptional program that alters cellular metabolism (117). Vascular smooth muscle and damaged endothelial cells undergo metabolic shift with reduced oxidative phosphorylation and glycolytic flux, which resembles Warburg-like behavior seen in other stress conditions (118). While glycolyssis may maintain ATP production at hypoxic periods, the long-term dependence is associated with mitochondrial dysfunction and redox imbalance (119).

Mitochondrial damage and loss of membrane potential are always accompanied by a higher level of reactive oxygen species (ROS) which besides their cytotoxic effects, serve as signaling molecules that sustain HIF-1α signaling, promoting hypoxia-related transcription (120, 121). Other metabolic intermediate components also display signaling functions. Lactate accumulates in local microenvironment via monocarboxylate transporters, and regulates the transcription of inflammatory genes via histone lactylation (122).

In general, hypoxia within aortic wall is not only an energy deficit issue but also a mechanism of coordinated reprogramming, in which the metabolic and transcriptional and inflammatory processes are highly complementary.

### 3.3 Metabolic-inflammatory coupling: formation of a nonlinear amplification network

The metabolic-induced disruption and accumulation of oxidative stress in vascular cells change the immune system of the aortic wall, linking metabolic sensing and innate immune activation. A key component of this network is the NLRP3 inflammasome, which integrates diverse damage-associated molecular patterns – including excess reactive oxygen species, mitochondrial DNA release, potassium efflux, and specific metabolites such as succinate and citrate. Mechanically, mitochondrial dysfunction and redox imbalance both promote NLRP 3 inflammaome assembly and activation in NF-κB-dependent transcriptional and post-transcriptional pathways connecting metabolic disruption to inflammatory effector responses.

The assembled NLRP3 inflammasome activates caspase-1, promotes the maturation and secretion of interleukin-1β and interleukin-18, and induces pyroptosis. This change marks the transition from transient stress adaptation to sustained inflammatory amplification within the vessel wall, leading to progressive tissue damage (123, 124).

The downstream cytokine cascade further alters the vascular environment. Inflammatory mediators like IL-6 stress endothelial activation, leukocyte attachment and migration, and also generate phenotype switching in smooth muscle (125). In the presence of inflammatory stress, smooth muscle cells can change from a contractile phenotype to synthetic, inflammation-inducing and senescence-associated phenotypes, where the number of chemokines, matrix-degrading enzymes and apoptosis-regulating molecules increases (126, 127). This plasticity of the phenotypes is harmful not only for structural stability but also contributes to immune cell recruitment, thus initiating positive inflammatory feedback loops.

Polarization in the aortic wall is strongly regulated by local metabolic state and oxygen availability (128). Hypoxia, HIF-1α stabilization and glycolytic reprogramming tend to favour polarisation to the pro-inflammatory M1 phenotype. M1 macrophages produce high levels of reactive oxygen species, nitric oxide, and matrix metalloproteinases which accelerate extracellular matrix degradation, while anti-inflammatory, reparative M2 macrophage, which rely on oxidative phosphorylation and fatty acid oxidation, are relatively scarce in diseased aortas, which is due to metabolic state-mediated restriction of immune tolerance (129, 130).

All these processes constitute a nonlinear amplification network. Hypoxia stimulates inflammation, and inflammatory signals stimulate mitochondrial dysfunction, oxidative stress, and metabolic instability (131). Such a combination exhibits a threshold-dependent behavior: as the increasing disturbances increase and become critical, the system would rapidly degenerate into irreversible tissue damage. Such system behavior can provide a theoretical explanation for the abrupt sudden catastrophic onset of aortic destruction (132).

We can think that targeting important metabolic elements (such as glycolysis, ROS production, and inflammasome activation) could break this amplification and restore normal vascular functioning. This is however difficult for cell-specific modulation without losing essential immune defense mechanisms.

**Figure 4:**
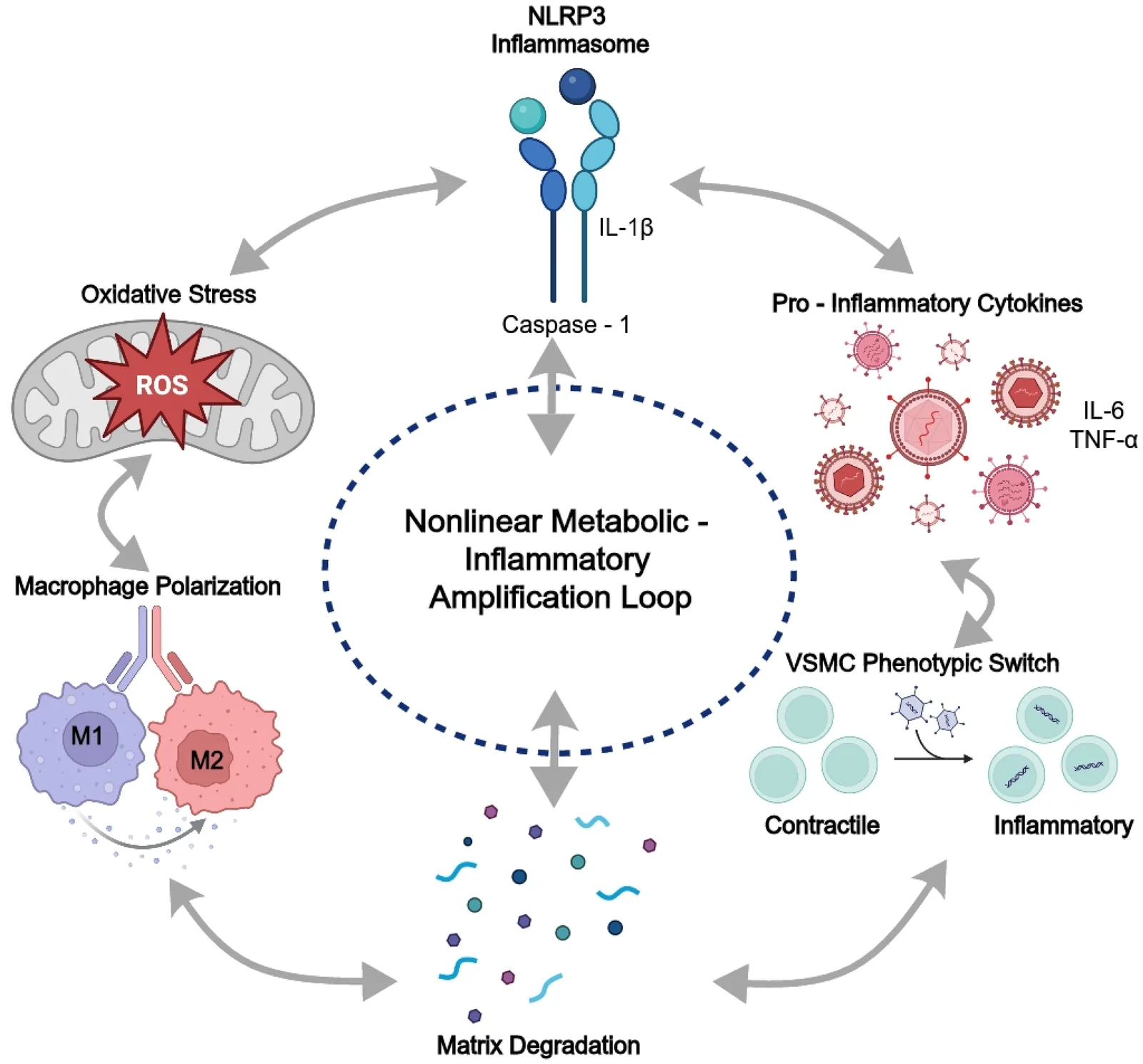
Nonlinear metabolic–inflammatory amplification network in aortic wall destabilization.

**Figure 4** depicts the nonlinear amplification network of this metabolic-inflammation coupling. As shown in the figure, the upstream microvascular dysfunction and hypoxia are the key initiating factors, which trigger a metabolic reprogramming centered on the stabilization of HIF-1α and the enhancement of glycolysis. This metabolic transformation not only alters the energy supply pattern of cells, but more importantly, it generates signal molecules such as reactive oxygen species, succinate, and lactic acid, which act as endogenous danger signals and directly activate the innate immune pathways represented by the NLRP3 inflammasome. Once the inflammasome is activated, downstream inflammatory factors such as IL-1β will form a positive feedback loop, further exacerbating oxidative stress and metabolic disorders, thereby forming a self-sustaining and continuously amplifying pathological cycle, pushing the local adaptive response towards structural destruction on a larger scale.

### 3.4 Iron metabolism and oxidative damage: a potential amplification pathway following micro-hemorrhages

Micro-hemorrhages of structurally degraded vasa vasorum drive erythrocytes into the media and the adventitia, triggering a recently underappreciated iron-dependent oxidative damage (133). The breakdown of hemoglobin releases heme and labile ferrous ions which catalyze Fenton reactions, leading to highly reactive hydroxyl radicals and greater local oxidative stress (134).

Iron-induced oxidative stress affects vascular smooth muscle cells. Intracellular iron overload increases lipid peroxidation and ferroptosis which involves oxidized phospholipids, glutathione depletion and glutathione peroxidase 4 (GPX4) (135). These activities not only contribute to the death and necrosis of smooth muscle, they also degrade their contractility and repair ability, and the structure of the aortic media becomes corrupted. Lipid peroxidation products such as malondialdehyde and 4-hydroxynonenal also act as danger signals and activate innate immune mechanisms such as the NLRP3 inflammasome (136, 137). This establishes a mechanistic link between iron metabolism and inflammatory amplification, which maintain the oxidative and inflammatory microenvironment even after the initial bleeding has subsided. Furthermore, iron transport proteins - ferritin, transferrin receptor and ferroportin - contribute to intracellular iron overload and tissue damage (138).

Clinically, such iron-dependent oxidative damage may drive progression from intramural hematoma to overt aortic growth. Repeated oxidative damage and loss of smooth muscle cells shrink the tensile strength of the media and increase the intimal tear and false lumen growth (139). The mechanistic explanation for the instability observed in some of the hematomas is mechanistic and demonstrates the importance of microvascular stability.

From a therapeutic point of view, strategies to control iron availability, scavenge free radicals, or enhance endogenous antioxidant capacity may have therapeutic benefit. Iron chelators, ferroptosis inhibitors and drugs for redox homeostasis would be of interest, but clinical safety of the drugs has not been assessed in the aortic diseases (140). In the future, iron imaging and molecular screening could assist in enhancing risk stratification and guiding personalized treatment strategies.

As shown in **Table 2**, there is a clear, cascading and amplified pathological chain from the initial pathological remodeling of the blood vessels to the eventual disintegration of the extracellular matrix during this process. Identifying the key molecules and driving mechanisms in this chain not only helps us understand the progression of the disease, but more importantly, it points out the potential clinical transformation directions for us, including developing new imaging monitoring methods, discovering liquid biopsy markers for early warning, and designing precise treatment strategies targeting specific pathological nodes. The following sections will further explore how these biological processes are confirmed by imaging and spatial multi-omics studies, and ultimately form a dynamic, non-linear amplification network.

**Table 2:** The vasa vasorum–inflammation–metabolism pathological axis: key molecular drivers and translational implications.

Axial linkage	Key molecules/markers	Pathological driving mechanism	Potential clinical translational significance
Initiating node (VV)	VEGF, HIF-1α	Microvascular permeability, local hypoxia	Anti-angiogenic therapy, microvascular imaging monitoring
Amplification center (metabolism)	Succinate, Lactate	Metabolic reprogramming, endogenous signal activation	Development of metabolic modulators, liquid biopsy biomarkers
Actuator (inflammation)	NLRP3, IL-6, MMP-9	ECM degradation, VSMC apoptosis	Precision immune blockade, risk stratification assessment
Structural manifestation (ECM)	Elastin Frag, Collagen	Collapse of mechanical scaffold, pseudocavity formation	Traditional surgical indications, mechanical strain assessment

### 3.5 Integrative evidence from imaging and spatial multi-omics

Mimodal imaging and molecular profiling with spatial resolution provides a complementary perspective to the clinical study of aortic decomposition. New technologies provide a direct way to study vascular pathology at spatial scales, from macroscopic structural changes to cellular and molecular heterogeneity.

Early MRI studies have already demonstrated that aortic dissection can occur without intimal tear, with the dissected lumen believed to represent intramural hemorrhage due to rupture of the vasa vasorum (141). High-resolution MRI, in combination with contrast enhancement and vessel wall sequences, can detect wall enhancement and specific patterns such as intimal flap and intramural hematoma in arterial dissection. Early MRI studies have already demonstrated that aortic dissection can occur without intimal tear, with the dissected lumen believed to represent intramural hemorrhage due to rupture of the vasa vasorum (142). The FDG-PET detects areas of increased metabolic activity in the aortic wall. These hypermetabolic foci reflect inflammatory cells entering the aortic region and higher glycolytic flux, and risk of structural degeneration and disease (143, 144).

Recent imaging techniques support this idea. Molecular imaging with respect to certain biological processes such as macrophage activation, matrix metalloproteinase activity, hypoxia signaling can reveal disease-related sites more precisely than conventional anatomical imaging (145). Overall, this suggests that metabolic-inflammatory “hotspots” in aortic wall are closely related to mechanical vulnerabilities.

Spatial transcriptomics and multi-omics-based methods preserve tissue organization can still be applied in a molecular level. Interacting inflammatory smooth muscle cell subpopulations, metabolically-regrogrammed endothelial cells, and infiltrating macrophages are not randomly interlocated, but co-localize in microanatomical regions of media and onset of aortic tissue (146, 147), in which hypoxia responses, glycolysis and inflammatory signalling are both activated simultaneously and are directly correlated with the vasa vasorum-hypoxia-metabolism-inflammation.

This spatial trend suggests that the pathology progresses not only in the wall of vessels, but also in focal pathological regions and the intimal rupture starts to develop in local areas. Single-cell sequencing and transcriptomic analyses also confirm that cell states in local regions are highly plastic and switch rapidly between contractile, inflammatory, and stress-responsive phenotypes depending on microenvironmental signals (148, 149).

Significant temporal support for this kind of scenario is also available from animal models. In various aortic aneurysm models, vasa vasorum blood flow, microvascular permeability and hypoxia-like activities like HIF-1α occur before major mechanical damage occurs, suggesting that initial microvortic remodeling and metabolic stress are early events and not secondary ones (150, 151).

There are several important open questions. The time-course relationship between microvascular dysfunction, metabolic reprogramming, immune activation, and structural damage is unclear, and observational data have not yet been able to provide reliable evidence of causal relationships. Longitudinal studies of serial imaging and spatial multi-omics in human cohorts as well as experimental models are needed to explore the relationship.

On the translational side, further imaging and spatial molecular data could improve risk stratification and early detection by allowing high-risk metabolism-inflammation microenvironments in the aortic wall to be localized by an accurate diagnosis before a fatal rupture, and by enabling aortic dissection to be treated precisely by linking local biology with a targeted therapy.

## 4 From molecular observations to system framework: multiscale coupling and causal structure of vessel wall destabilization

One of the primary theoretical issues in aortic dissection research is to conceptualize disease. Several studies have reported individual pathological changes, such as endothelial abnormalities, phenotype switching of vascular smooth muscle cells, sustained erosion of the extracellular matrix, and remodeling of the vasa vasorum. Yet it is not clear what role these changes play – for example, how they relate to each other and to disease progression (152–154). This is in sharp contrast to the fact that chronic hypertension is very common for most of the population, but aortic dissection is rare – we have a strong intuition that mechanical stress alone leads to disease onset (155). Most of the patients with hypertension maintain the structural integrity of the aorta, and many do, however, collapse during the course of the study.

These functional modules normally operate in a coordinated manner to maintain vascular homeostasis (156, 157). A limited perturbation to one module can often be buffered by the compensatory capacity of the others, such that the vessel wall tolerates a certain degree of stress without immediate structural compromise. However, when multiple modules become simultaneously dysfunctional—for instance, when endothelial mechanotransduction, smooth muscle cell phenotype control, and microvascular support all deviate from their normal states—the vessel wall may progressively lose its ability to withstand mechanical and biochemical stress, even while gross structural integrity appears largely preserved. If pathological positive feedback loops become established between these failing modules, the system can be driven toward a critical mechanical state. Once this point is crossed, structural failure, such as delamination or tear propagation, becomes inevitable (158, 159).

### 4.1 Multiscale coupling: a nonlinear understanding of vessel wall destabilization

The most conventional description of aortic dissection is mechanical: when there is a large stress on the aortic wall, blood enters the media (160). This represents the final event, but it does not include previous molecular and cell pathological changes. Both cytochemical and advanced imaging studies consistently indicate that a significant period of structural weakness precedes clinical rupture. In areas of dissection that occur later, subtle abnormalities such as mild inflammatory infiltrates, elastic lamellar fragmentation, and dilation of adventitial microvessels are often detected before the intimal tear is observed (161).

These results suggest that aortic rupture is a sudden unpredictable event, but a gradual transition to a structurally unstable state (162). From a network regulation perspective, the aortic wall may be regarded as a system of multiple interacting regulatory nodes, including endothelial mechanosensors, transcription programs for smooth muscle cell phenotype, extracellular matrix (ECM) remodeling or the metabolic microenvironment maintained by the vascular supply system (163). The regulators communicate with each other via mechanical signals, cytokines, reactive oxygen species and metabolic mediators. The small perturbations inside a module are controlled locally, but for more than one node, the compensatory capacity falls sharply (164, 165).

According to complexity theory, some types of very dynamic networks can exhibit critical slowing down where their recovery rate from perturbation gradually declines and regulatory signals become increasingly erratic. Remarkably, transcriptomic analyses of diseased aortic tissue have identified molecular signatures that phenotypically resemble this kind of network-level destabilization. A recent multi-omics study showed strong upregulation of inflammatory programs along with widespread dysregulation of homeostatic gene regulatory networks in aortic dissection, which although static snapshots cannot capture the dynamics of critical slowing down, offer an intriguing molecular background for it. These findings suggest that there might be a slow destabilization of the regulatory network of the aortic wall conceptually similar to critical slowing down long before the abrupt mechanical tearing takes place (166, 167).

#### 4.1.1 The functional triad: endothelium – smooth muscle – matrix

The structural homeostasis at the aortic level depends on the precise coordination between endothelial cells, vascular smooth muscle cells, and the extracellular matrix, which are a functional three-part combination in which mechanosensing, cellular adaptation, and matrix maintenance are interdependent and inseparable (168).

Hemodynamic signals are converted into transcription programs that maintain the vasculature. If mechanosensory pathways are degraded, this regulation is disrupted (169). Lower activity of protective transcription factors and a lack of nitric oxide signaling lead endothelial cells to an inflammatory phenotype and cytokines and chemokines that affect media and the environment of smooth muscle cells are released (170).

Smooth muscles display surprisingly fine-grained plasticity. Under the influence of inflammatory mediators and oxidative stress, contractile genes are suppressed, and programs involving matrix remodeling and cell migration are induced. One consequence is increased production of matrix metalloproteinases degrading elastic fibers and collagen, progressively degrading the mechanical elasticity of the vessel wall (171, 172).

This is not only a structural loss. Because the peptide fragments produced by elastin degradation act as internal danger signals that trigger inflammation, the matrix degradation in turn amplifies the first inflammatory microenvironment and causes a closed regulatory loop. The endothelial dysfunction produces inflammatory signals, the inflammatory signals reprogram smooth muscle cells, the reprogrammed smooth muscle cell accelerate matrix degradation and the matrix degrading amplifies inflammation (173). This feedback loop increases over time and progressively degrades the structural reserve of the aortic wall (174).

#### 4.1.2 The adventitial microvessel-metabolic regulatory axis

Although the three functional factors play a particular role in the wall of the media, another important role arises from the birth of the vasa vasorum. In the physiological condition, the vasm vasorum carries oxygen and nutrients to outer layers of the aorta, but in the pathological condition, this microvascular network is often expanded and remodeled (175).

Does the remodeling biological significance? Young vessels with immature structure and basement membrane defects can suffer from micro-hemorrhages and local hypoxia. Insufficient oxygen supply promotes long-term expression of the transcription factor HIF-1α altering the transcription profile of surrounding vascular cells; common effects include activation of glycolytic pathways and suppression of mitochondrial oxidative phosphorylation (176, 177).

The metabolic processes that arise in such conditions can influence immune response. Succinate affects macrophage polarization; succinate increases hypoxia signaling and inflammatory transcription (178). Metabolomics of aortic dissection tissue often contain higher levels of glycolysis metabolites that correlate with inflammatory markers. These suggest that microvascular remodeling can lead to hypoxias, metabolic response and immune activation that are overlapping with the endothelium-smooth muscle-matrix triad described above, allowing metabolic perturbations in the outer layer to propagate inward and impair medial homeostasis (179, 180).

### 4.2 Temporal progression of vessel wall destabilization

For us to understand the events before rupture, we need to take into account their behavior over time. Structural failure typically occurs suddenly, and only when a critical transition point is reached and compensatory mechanisms have been exhausted (181).

Small changes in homeostasis occur at an early stage of disease. Compensatory remodeling occurs within the adventitia; subtle shifts in metabolic flux and inflammatory signals take place despite a largely intact gross extracellular matrix structure (182). This stage has enough feedback mechanisms that there appears to be homeostasis.

Next, there is an subsequent period of inflammation and macrophage invasion. MMP activity exceeds that of the endogenous inhibitor and matrix decomposition accelerates. The elastic lamellae are fragmented and macroscopic structures are preserved. At each stage the vessel wall becomes mechanically critical. Layers of elastic fibers creat localized stress sites, and the biomechanical model of the vessel indicates that the peak stress is high. In this state even a small change of blood pressure could produce a dramatic structural response (183–186).

The end is interrupted by an intimal tear, blood pumping into the media and a false lumen – the classical clinical result of aortic dissection – as molecular abnormalities become mechanical decompensation and the process accelerates rapidly (187). These progressive phases – from early compensatory shifts, through inflammatory amplification and structural criticality, to overt mechanical failure – are systematically summarized in **Table 3**, which provides a temporal framework for understanding the nonlinear decay of aortic wall homeostasis.

**Table 3:** Temporal stages of aortic wall homeostatic erosion: from subclinical shift to clinical catastrophe.

Evolutionary stage	Core pathological features	Representative molecular/imaging markers	Steady-state evaluation
Substeady-state shift	Increased VV density, metabolic fine-tuning	HIF-1α (↑), weak PET uptake	Compensatory steady state
Inflammatory amplification	Macrophage infiltration, MMP activation	IL-6 (↑), MMP-9 (↑)	Instability initiation
Structural criticality	Elastic fiber rupture, stress concentration	EDPs (↑), HR-MRI signal enhancement	Critical state
Clinical manifestation	False lumen formation, intimal-media separation	CTA showing true/false lumen	Steady-state collapse

### 4.3 Evidence from single-cell and spatial multi-omics analysis

Large-scale molecular profiling can begin to uncover the cellular structure of these processes. Single-cell RNA sequencing studies found multiple smooth muscle cell subpopulations in aortic dissection tissue, where inflammation-related genes showed high expression and reduced contractile function (188). Additional insights from multi-omics analysis on the interaction between metabolism and immunity. Transcriptomic and epigenomic profiling demonstrated positive correlation between glycolytic signatures and inflammatory genes as well as macrophage infiltration markers in aortic dissection tissue (189, 190). Convergence of metabolic dysregulations and immune activations suggest their independence is broken by being mechanistically linked together in the vessel wall.

Further evidence supporting an integrative perspective has been obtained through transcriptomic network analysis. Genes associated with metabolic regulation and inflammatory signaling consistently appear as hubs within weighted gene co-expression networks constructed using aortic dissection tissues; thus, they act as key orchestrators of the transcriptional landscape in the diseased vessel wall (191, 192). The topological structure suggests that metabolic and immune modules are not peripheries but deeply rooted in the regulatory framework maintaining or losing vascular homeostasis.

### 4.4 Experimental evidence supporting causality

Future reliability of the system model will ultimately depend on whether the predicted causal relationships can be verified experimentally. Animal studies are also beginning to partially support some parts of the model.

Progressive angiogenesis inhibition reduces vasa vasorum density and dissection incidence in animal models, suggesting that microvascular remodeling plays an upstream role in the pathological pathway (193). By inhibiting glycolytic activity of smooth muscle cells, we may partially restore the contractile phenotype and suppress inflammatory signals, indicating that metabolic reprogramming is actively driving in phenotype switching (194) while experimental inhibition of inflammatory pathways slows down the process of extracellular matrix degradation (195).

Although these results are do not prove that vessel wall destabilization is fully reversible, they still indicate that the network of aortic structural integrity can be a little plasticized in some form before a critical threshold is reached. Understanding this reversible window will be critical for future prevention of rupture in high-risk populations (196, 197).

**Figure 5:**
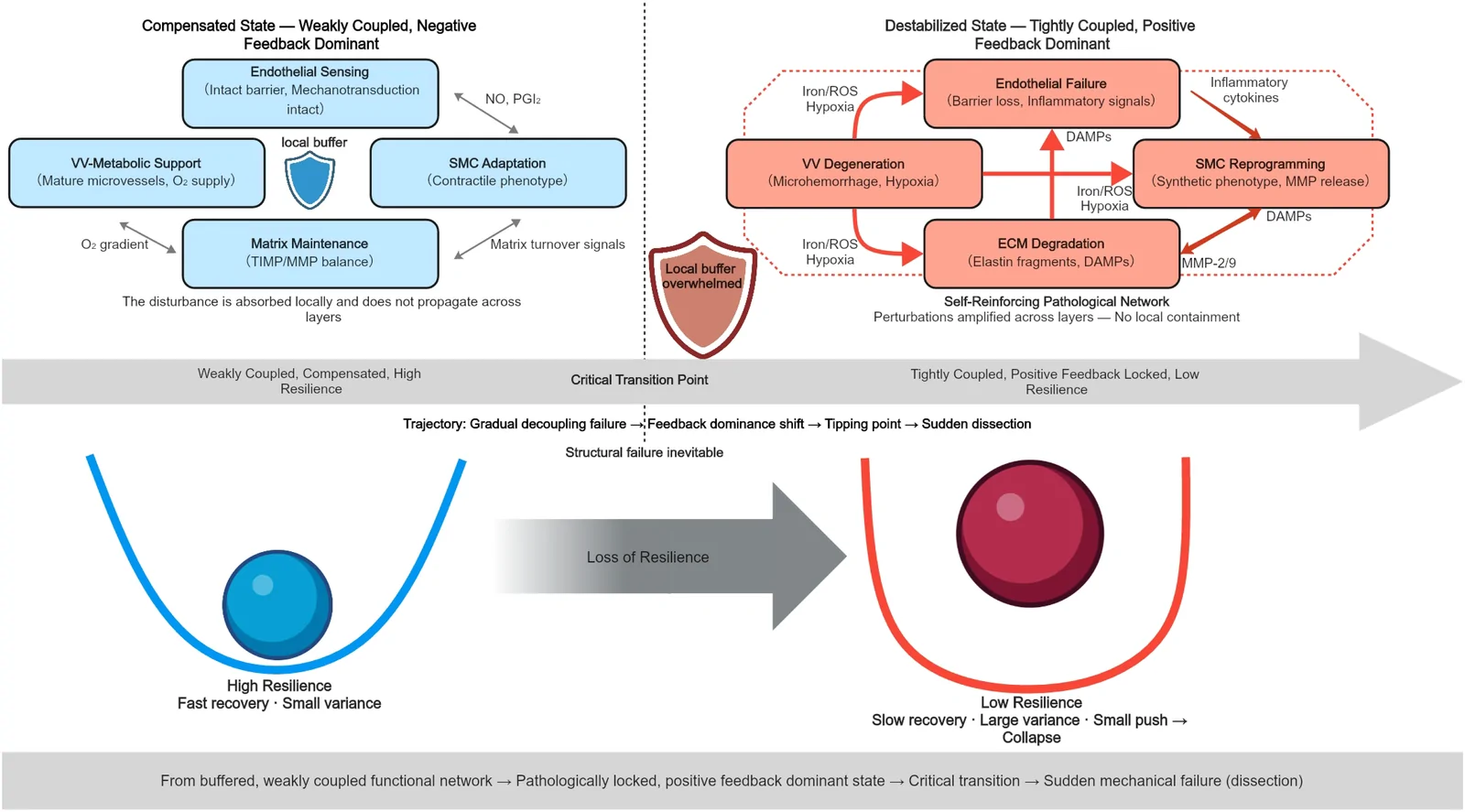
Multilevel coupling and nonlinear causal architecture of aortic wall.

In order to integrate the pathological processes of such interactions, we present a schematic diagram (**Figure 5**). Instead of focusing on isolated roads, we take into account the aortic wall as a connected functional network consisting of endothelial sensing, smooth muscle adaptation, matrix maintenance and adventitial microvascular-metabolic supports, which emphasizes the transition from a weakly coupled, compensated to a positive feedback and low robustness state. By incorporating spatial structure and time flow, we show that perturbations without early clinical signs emerge between layers, gradually advancing the system towards a critical point. In this sense, overt aorti dissection is treated as a sudden mechanical collision, which is viewed as the end-phenotype of progressive destabilization of the regulatory network.

In the compensated state, the four functional modules of the aortic wall — endothelial sensing, SMC adaptation, matrix maintenance, and VV-metabolic support — are weakly coupled through negative feedback-dominant signals. Local perturbations are buffered within individual layers and do not spread. During destabilization (right), VV dysfunction, endothelial barrier breakdown, and SMC reprogramming drive a shift toward tight, pathological coupling. A self-reinforcing network emerges: DAMPs from the matrix, inflammatory cytokines from dysfunctional endothelium, MMPs from synthetic SMCs, and iron/ROS/hypoxia from degenerated VV amplify each other across layers. This transition is marked by progressive loss of resilience (potential well shallowing, bottom), critical slowing down, and increased variance. At the tipping point, even a minor hemodynamic perturbation can trigger the sudden mechanical collision of aortic dissection — the end-phenotype of gradual network destabilization. VV, vasa vasorum; SMC, smooth muscle cell; ECM, extracellular matrix; DAMP, damage-associated molecular pattern; MMP, matrix metalloproteinase; ROS, reactive oxygen species. .

## 5 Clinical evidence and quantitative indicators: translating the vascular homeostasis theory into clinical practice

The theory of aortic dissection that could explain the underlying causes must eventually be validated in practice. Namely, could their fundamental ideas translate to observable and testable clinical characteristics? Mechanistic plausibility cannot be adequate; for a framework to be generalizable, the underlying biological processes would have to appear as trackable, measurable phenotypes of living patients and have to be monitored. The vessel wall homeostasis imbalance theory raises the question: if abnormal endothelial signaling, metabolic dysfunction, inflammatory activation, or immune reaction lead to abnormal structural damage, what can be accomplished? (198, 199).

Classic diagnosis of aortic disease relies on anatomical parameters.The diameter of the aorta, increase rate, and intimal tear are currently the primary criteria for surgical treatment (200). However, clinical registry studies show that a significant fraction of patients with aortic disease did not meet the classic criteria for prophylactic repair preoperatively, suggesting that significant anatomical expansion is not a necessary prerequisite for dissection, and thus structural measurements capture the endpoint, not the origin. Structural measurements capture the endpoint, not the origin (201).

This has raised the point that clinical thinking is not treating anatomical changes as the sole risk signal, but rather as a reflection of the degree of functional disturbance in the regulatory networks keeping blood flow healthy. Abnormal microvascular effusion of the vessel wall, chronic inflammatory response and hypoxia-mediated metabolic changes together are early functional markers of vessel wall instability (202, 203). This difficulty is to translate these pathological processes, which are usually studied at the molecular level, into clinically meaningful indicators representing aortic wall dynamics (204).

### 5.1 Imaging evidence: multimodal visualization of microvascular destabilization and inflammatory activity

Cardiovascular imaging is becoming increasingly sophisticated for detecting abnormalities in the aortic wall. While classical imaging was primarily concerned with reconstructing luminal anatomy, recent advances focus more on the biological activity of the vessel wall. High-resolution black-blood magnetic resonance imaging, which suppresses intraluminal blood signals and enhances contrast within the vessel walls, can be used to visualize subtle medial changes – small intramural hematomas, focal edema, or discontinuities of elastic lamellae, even when luminal morphology is usually normal (205–207).

The pathological characteristics of the early microcirculation within the outer aortic wall should also be noted; in late stage dissections, diseased microvessels have been observed as being rapidly growing and structurally immature with basement membrane deficiencies and weak endothelial junctions – which is thought to be driven in part by endothelial-to-mesenchymal transition; while it may be difficult to directly image the aortic vasa vasorum, these structural deficits will lead to increased microvascular permeability resulting in leaking of plasma components into the surrounding adventitial matrix.

Such imaging measures cannot be viewed as a larger microvascular volume. Permeability changes and diffusion effects may be more important because both contribute to cytokines and reactive oxygen species accumulation in the newly born microenvironment. Continuous exposure to medial smooth muscle cells increases the changes of their transcription programs, linking microvascular dysfunction to structural loss of vessel wall (208, 209).

An additional information dimension is functional metabolic imaging. Positron emission tomography (PET) with glucose tracers can determine areas of increased demand (210, 211). Immune-activating inflammatory cells like macrophages and neutrophils often activate by glycolysis; therefore, higher tracer uptake is frequently observed in inflammatory invasion regions (212). The metabolic signals may sometimes be detected before structural imaging reveals damage to elastic fibers.

The spatial relationship between metabolically active inflammatory sites and the aortic wall is being studied. Some observational studies suggest that focal metabolic “hotspots” in the aortic wall may coincide with areas which are damaged later (213, 214). The causal relationship remains unclear, but this suggests that metabolic imaging may capture a transition point during which tissue structure is intact but functional stability is still limited.

In addition to image interpretation, studies of imaging data are also revealing more subtle pathological patterns. Radiomics may quantify textural variability, signal gradients and spatial complexity in images. These mathematical features may reflect the underlying tissue changes – collagen configuration, microvascular distribution and cell density – that are unobserved by the eye. If validated, radiomics signatures could be macroscopic “fingerprints” of the tissue state of the vessel wall. Risk testing would then switch from relying solely on a single imaging parameter, to a multi-dimensional test of dysregulation between structure and function in the aortic wall (215, 216).

**Table 4** summarizes these advanced imaging techniques, whose clinical value goes far beyond merely observing the structural damage at the end stage. What is more important is that they have for the first time provided us with the possibility of non-invasively “inspecting” the biological state of the aortic wall in a living body. By jointly applying these modalities, clinicians are expected to shift from a single “anatomical diameter” assessment to a more comprehensive “biological risk” stratification, identifying high-risk patients at an early stage before irreversible damage to the structure occurs, thus achieving true early warning and intervention.

**Table 4:** Multimodal imaging techniques for assessing aortic wall biological status: beyond anatomical surveillance.

Technology	Measurable indicators	Clinical value
MRI	Wall thickness/signal	Early detection
PET	FDG uptake	Inflammatory activity
CTA with contrast	microvascular perfusion	Risk stratification

### 5.2 Circulating biomarkers: systemic molecular signatures of vessel wall destabilization

While the imaging reveals local tissue changes, circulating biomarkers could also capture molecules released by the pathological processes, and could be a “liquid biopsy” of the aortic wall. Events such as extracellular matrix breakdown, immune cell activation, and hypoxic adaptation, all produce molecular fragments entering the blood (217, 218).

Inflammatory cytokines are the most widely studied class of markers. However, markers such as interleukin-6, C-reactive protein and tumor necrosis factor-α are not disease-specific and may be elevated in other inflammatory conditions. Nevertheless, longitudinal monitoring studies have shown that their dynamic trajectories are linked to disease progression, and a sustained elevation may reflect ongoing immune activation within the aortic wall (219).

Elastin degradation fragments are a class of proteins that are directly structural related. Elastin is important for maintaining elastic recoil in the artery and degradation signals damage to the structural support structure. Elastin peptides can not only be products of structural damage, but can also be involved in biological activity: some may serve as endogenous danger signals, activate innate immune receptors on macrophages, and activate inflammation within the vessel wall due to structural damage (220–222).

Matrix remodeling enzymes can also be understood in dynamic tissue metabolism. Higher levels of matrix metalloproteinase 9 (MMP-9) tend to drive vascular remodeling. It has been observed that elevated metalloproteinase activity occurs before medial rupture before medial rupture of the media occurs, showing that enzymatic degradation of matrix components can be considered as a key step in the transition from inflammation to structural failure (223, 224).

Metabolic markers add another measure of vascular risk. Metabolomic profiling studies have shown that circulating levels of glycolysis-related metabolites, such as lactate and succinate, are associated with cardiovascular disease risk, suggesting that these metabolites may reflect local metabolic reprogramming within the aortic wall (225). Bioinformatics analyses of aortic dissection tissues have further identified HIF-1α as a hub gene within the dysregulated molecular network, linking hypoxia-responsive metabolic shifts to inflammatory signaling pathways (226).

There is no single class. Biologic variability, transient systemic inflammation and assay variability, could hide the clinical significance of a single measure, but when markers from multiple aspects – immune activation, matrix degradation, metabolic dysregulation – are considered together they usually have a predictive value. High markers from several domains could be believed to indicate that some of the regulatory subsystems in the vessel wall have not been homeostatic.

**Figure 6:**
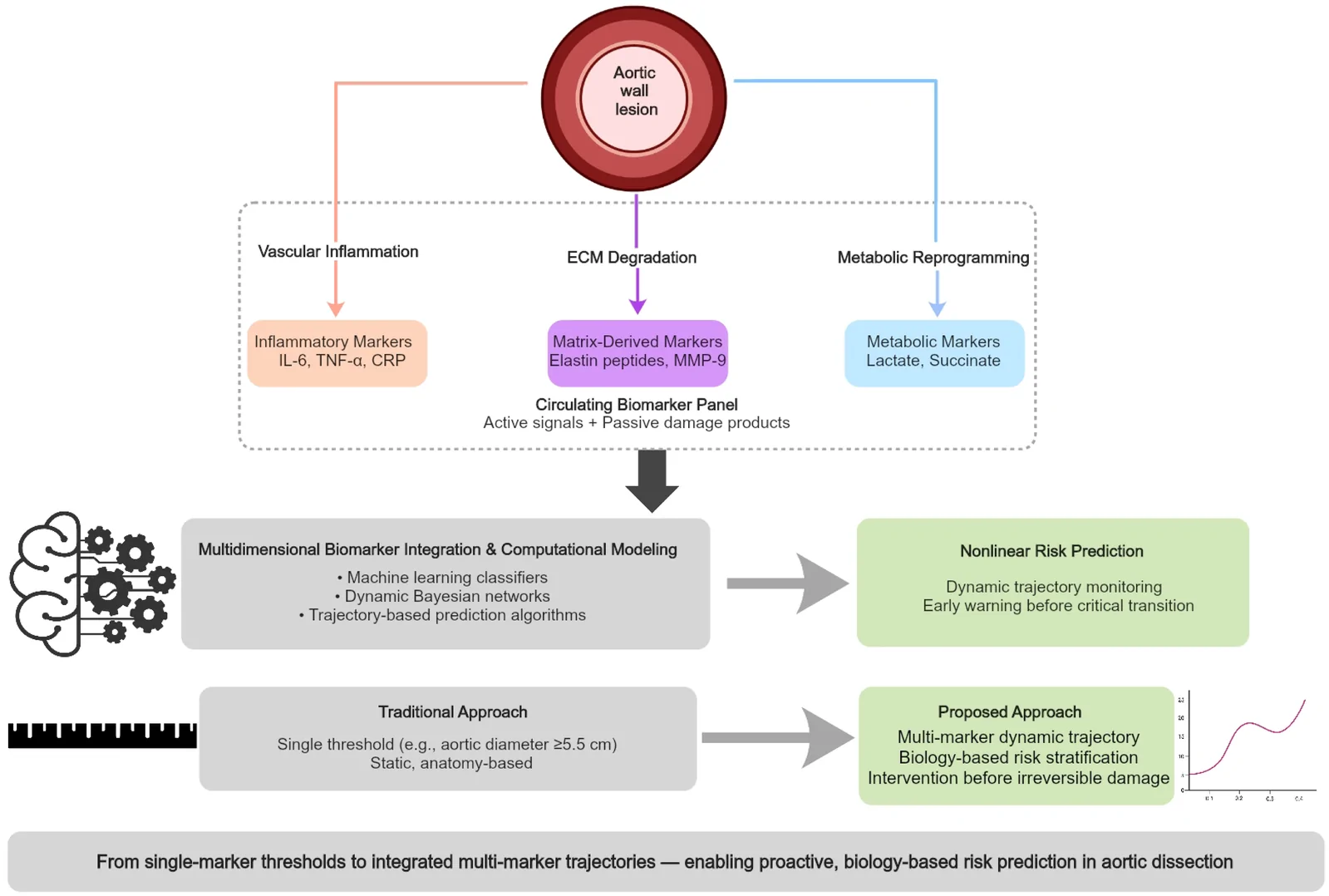
Integrated circulating biomarker network and nonlinear risk prediction framework in aortic dissection.

In order to capture these findings, we present a schematic diagram (**Figure 6**) between local pathological changes in the vessel wall, systemic biomarker profiles and downstream risk prediction. The diagram shows how vascular inflammation, matrix decay, and reprogramming within the aortic wall generate local but connected molecular signals of circulating blood, and that the clinical utility of these markers is not limited to individual test results, but in their integrated interpretation in a multidimensional setting. This can be combined with computational modeling and potentially further lead a shift from linear risk assessment to nonlinear, trajectory-based prediction, and circulating biomarkers are also passive indicators of damage, and active signals of changes in systemic homeostasis of the vasculature.

### 5.3 Risk prediction models: from linear statistics to nonlinear system dynamics

The method to interpret clinical data may inform the conclusions drawn. Risk models typically rely on statistical regression based on a rather stable relationship between predictors and results. For sudden transitions, this may be an inaccurate assumption. The course of aortic dissection is similar to a threshold phenomenon: prolonged stability lasts until sudden structure collapse (227).

Complexity theory describes a strikingly similar pattern. Many nonlinear systems follow a similar pattern: across a constant range of control parameters the system remains stable until a critical value is reached, at which time it abruptly moves towards a new value (228, 229). Drawing on this framework, our interpretation of vascular biology suggests that progressive weakening of homeostasis, even without significant anatomical changes, eventually leads to mechanical collapse.

Machine learning methods are increasingly used to learn these patterns. Algorithms which can analyze high dimensional clinical data can incorporate imaging features, laboratory conditions, and demographic variables in order to predict disease events. Deep learning models, particularly, are able to capture nonlinear relationships that are not possible with traditional statistical methods.

Another persistent problem is model interpretability. Prediction models referred to as “black boxes” are not useful for clinical decisions. Exploiting explainable artificial intelligence models can better assess the role of each variable on a final prediction to see if the inflammatory markers, microvascular signals, or metabolic signals play an important role in the decision making in a model.

It may be desirable to move away from static prediction and toward dynamic prediction of disease trajectories. For example, dynamic Bayesian networks could model the behavior of the vascular system in different environments, mapping possible paths of disease evolution that extend beyond a single probability estimate. Such a trajectory prediction may reflect biological reality as the vessel wall continuously adapts to mechanical and metabolic stimuli (230–232).

### 5.4 Translational implications: restoring vascular homeostasis in the era of precision medicine

If vessel wall homeostasis is indeed maintained by multiple interacting regulatory processes, therapeutic strategies targeting a single pathway may have limited efficacy. Intervention methods involving one molecular process may not be able to stabilize the whole network well, but combining approaches of several components – endothelial signalling, immune activation, matrix metabolism, cellular metabolism, could be beneficial in restoring balance (233, 234).

Longitudinal monitoring of biomarkers of these processes is promising for early detection of individuals in whom vascular homeostatic regulation is beyond the normal range, theoretically allowing intervention in an intermediate stage, before irreversible structural damage is done, but concrete treatment methods could not yet be proposed. Experimental works show potential to regulate inflammatory signaling, metabolism and microvascular remodeling by pharmacological effects, but their translation into clinical practice has not yet been realized (235, 236).

More generally, the homeostasis concept suggests a paradigm shift in how aortic disease can be treated. Aortic dissection should not be an isolated catastrophic mechanical event but the result of the inevitable cascade of regulation functions at various biological scales. Molecular biology, advanced imaging, computational modeling and clinical observation are beginning to reveal the relationships between these scales, potentially ultimately changing the way aortics is treated from acute crisis intervention to proactive prevention based on quantitative signs of vascular homeostatic status.

In addition to biomarker-guided risk stratification and early detection, the vascular homeostasis framework has immediate prognostic relevance in patients who undergo aortic surgery—clinical evidence accumulates validating that inflammation persists (even after the diseased segment is resected) shaping long-term outcomes.

A retrospective analysis on patients who underwent the Bentall procedure found no impact from the presence of histological evidence for aortic inflammation on their postoperative survival but greatly elevated the risk of reintervention in the distal native aorta (Tarone-Ware test p < 0.01), with an important message: replacing the proximal segment by surgery may be lifesaving, but it does not remove the biological vulnerability of the remaining aorta; the inflammatory milieu which predisposed the original segment to degeneration remains present in the residual native aorta promoting progressive wall deterioration leading to eventual distal reintervention (237). Interestingly, this phenomenon goes further than the Bentall procedure—we observed similar findings after aortic arch surgery as well as after valve-sparing procedures, indicating that inflammation represents a constant driver of adverse aortic remodeling irrespective of the index operation.

It is also supported by a multicenter study of noninfectious surgical thoracic aortitis showing that 82% of patients have a vascular complication within 10 years; certain histopathological and clinical features identify high-risk patients who require subsequent vascular interventions (238). Subsequent analysis of this cohort showed that 31.5% of these patients died within 10 years after their initial surgery due to decreased survival attributed to early or late aortic dissections. The effect of inflammation on postoperative outcomes goes beyond aortitis cohorts as well: Admission inflammatory markers including interleukin-6, C-reactive protein, and composite indices are independently associated with postoperative complications, prolonged intensive care stay, and long-term survival in acute type A aortic dissection patients. IL-6 specifically predicts postoperative negative remodeling of the residual descending aorta likely through perpetuation of inflammatory cell infiltration and wall degeneration.

Classification and therapy: In his large surgical series (n = 156) on patients with histologically confirmed aortitis, Svensson et al. highlighted how our current classification of inflammatory aortic disease – encompassing giant cell aortitis, Takayasu arteritis, and nonsyndromic aortitis is too crude to inform targeted treatment and called for better histopathological characterization so we can pursue more specific therapies (239). Within the vascular homeostasis framework described herein, inflammation is no longer viewed as something which needs to be ‘resected’ or ‘suppressed’, rather it drives multi-system destabilization – affecting endothelial function, smooth muscle cell phenotype, extracellular matrix integrity, and vasa vasorum permeability in an interconnected, self-amplifying fashion.

Thus, achieving vascular homeostasis requires not just surgical correction, but also isolation of one mediator is insufficient—it calls for an integrated strategy combining: (i) timely surgery for hemodynamically significant lesions with intraoperative histology to guide surveillance intensity; (ii) continuous postoperative monitoring of inflammatory and metabolic biomarkers to identify early signs of distal remodeling; (iii) mechanism-targeted adjunctive therapy targeting either the NLRP3 inflammasome, metabolic reprogramming, vasa vasorum stabilization—or, in systemic vasculitis, immunomodulatory agents—to target upstream drivers of wall destabilization. Clinical evidence from Bentall and aortitis cohorts supports this unifying concept: Surgery fixes the tear, while homeostasis-focused management secures the long-term fate of the aorta.

### 5.5 Relationship between the framework and current clinical guidelines: complementarity, not negation

The integrative framework of vascular wall homeostatic imbalance provides us with a new theory that aortic dissection is not just an acute structural failure; it’s the terminal manifestation of protracted homeostatic deterioration. But there arises another critical question—If the intimal tear is simply the “terminal manifestation”, what about our current surgical strategies focused on “repairing the intimal entry” (ascending aorta replacement, total arch replacement, TEVAR, etc.) as being only “palliative”? Does this framework change the emergency surgical indications in acute Stanford type A aortic dissection?

#### 5.5.1 Emergency surgical indications: the framework does not alter them

Unquestionably, according to the 2022 ACC/AHA guidelines, urgent surgical repair should be considered a Class I indication for acute Stanford type A aortic dissection (ATAAD), given its immediately life threatening nature; without surgery, the mortality rate of ATAAD is 1-2% per hour during the first 24 hours after symptoms occur with up to 50% by 48 hours and 50-91% by one week (240). According to IRAD data, in hospital mortality is 18% among surgically treated patients compared to 52% when managed non-operatively (241). Whether we consider it “pure mechanical tearing” or “terminal manifestation of protracted homeostatic deterioration”, the immediate lethality of ATAAD is the same regardless of what caused it. Our framework will not change any current emergency surgical indications.

#### 5.5.2 Achievements and limitations of surgery

Recognizing the need for emergency surgery is not synonymous with ignoring its shortcomings. The accumulation of long-term follow-up data reveals a critical fact: while surgery fixes the proximal aorta, it leaves behind the biological vulnerability of the residual aorta.

A study of 225 patients after ATAAD repair reported event-free survival rates of 82%, 72%, 48%, and 33% at 1, 5, 10, and 15 years postop, respectively—that is, at 15 years post-surgery, less than one third of patients have avoided aortic events requiring reintervention (242); residual aortic dissection was found to be an independent predictor of reintervention (OR = 3.29) and 68% of patients following ATAAD repair still had persistent dissected intimal flaps (243, 244). This clearly demonstrates that although surgery is life saving, it is not curative—it excludes the entry tear, restores true lumen perfusion, and prevents cardiac tamponade and sudden death—achieving at the “life-saving” level, far from being “palliative,” yet unable to reverse the pathological processes already started on the residual aortic wall—the persistent inflammatory microenvironment, VSMC phenotypic dysregulation, extracellular matrix proteolytic imbalance, and vasa vasorum dysfunction.

#### 5.5.3 Inflammation: the key bridge connecting the framework to clinical prognosis

We stress on the importance of the inflammation-metabolism-vasa vasorum axis for the pathogenesis of dissection; importantly, it remains true during the postoperative period too. A meta-analysis including 79 studies showed that survivors have significantly lower white blood cell count, neutrophil count, neutrophil-to-lymphocyte ratio, and C-reactive protein than non-survivors (245). The preoperative pan-immune inflammation value (PIV) independently predicts prolonged intensive care unit stay and postoperative complications (246). Significantly, we found that interleukin-6 (IL-6) predicted negative remodeling of the descending aorta after ATAAD repair—incidence of negative remodeling at 2 years post-surgery was 24.0%; the negative remodeling group has significantly higher follow-up mortality (8.0% vs 1.3%), reoperation rate (10% vs 1.9%). Increased level of IL-6 is an independent risk factor (OR = 3.910); thus validating the main prediction of our framework – inflammation being both an upstream driver of dissection occurrence AND a persistent driver of residual aortic deterioration postoperatively (247).

#### 5.5.4 Three levels of complementary value of the framework to guidelines

Our integrative framework does not compete with or negate the current ACC/AHA guidelines; rather, it complements and deepens them in three ways:

First, consensus in the management during the acute phase. This framework will not change the guidelines’ recommended emergency surgical indications. Surgery is the only intervention that can change the fatal outcome during the acute phase of ATAAD.

Second, mechanistic rationale for post-operative surveillance. Guidelines recommend lifelong imaging surveillance after ATAAD repair; however they lack a sufficiently mechanistic basis as to why this intense monitoring needs to be performed despite successful surgery. We have provided an answer: Even after successful proximal aortic replacement there remains a homeostatic imbalance within the residual aortic wall. Inflammatory markers remain elevated, hemodynamics of the false lumen persist, aberrant vasa vasorum remodeling – these biological events do not cease upon surgery. This offers a robust pathophysiologic basis for the guideline recommendation of lifelong surveillance.

Third, guidance for future therapeutic strategies. Our framework highlights new directions: adjunctive therapies targeting the inflammation-metabolism-vasa vasorum axis – such as anti-inflammation, metabolism or vasa vasorum stabilization – may be potential ways of reducing long-term reintervention rates in addition to surgery alone. The fact that IL-6 predicts negative remodeling implies that inclusion of inflammatory biomarkers in postoperative risk stratification can lead to improved individualized surveillance strategies.

We do not change any guideline recommendations regarding emergency surgical indications; we transform how we understand what surgery achieves versus fails to achieve: It saves lives – excluding the entry tear, restoring blood flow, preventing immediate death—but surgery is not curative—the biological vulnerability of the remaining aorta remains, as well as dysregulation of the inflammation-metabolism-vasa vasorum axis that exactly explains the persistently high rate of long-term reintervention. Our framework provides a mechanistic rationale for guideline-recommended lifelong surveillance, novel biomarkers (inflammatory indices) for postoperative risk stratification, and future adjunctive therapies beyond structural repair only. This framework is not an attack on current guidelines, but a deepening and complement to them—a shift from managing aortic dissection with “one-time structural repair,” towards a new paradigm of “lifelong homeostatic maintenance”.

## 6 Translational pathways: from mechanistic analysis to “vascular homeostasis restoration” strategies

Aortic dissection is traditionally considered as a critical structural collapse of the aortic wall. Therefore, clinical treatment focuses on hemodynamic stabilization or emergency surgical or endovascular repair (248). Evidence from clinical imaging indicates that the aorta may contain subclinical structural abnormalities prior to overt dissection. In fact, CT has incidentally detected asymptomatic, subclinical thoracic aortic dissections in patients being surveilled for other aortic pathologies suggesting there is a clinically silent period of disease progression (249).

The phenotype of vascular smooth muscle cells, metabolic stresses, and the establishment of a chronic inflammatory zone can precede significant anatomical changes (250). These findings raise a fundamental question: Is aorta dissection simply a mechanical event or a critical transition driven by the continuously destabilizing process of a complex biological system? From the perspective of systems biology, the aorta is a dynamically regulated multiscale network of endothelial cells, smooth muscle cells, adventitial fibroblasts and immune cells exchanging mechanical, metabolic, and inflammatory signals continuously. When the total mechanical load, inflammatory effects, and metabolic disruptions reach a critical value, the stability of the system deteriorates further. Excessively buffered perturbations can result in structural collapse.

Building on this framework, some therapeutic actions should be towards recovering a stable state of the anatomy, for reinstating the capacity of vessel wall homeostasis to absorb, adapt to, and dissipate external disturbances (251). However, restoring homeostasis would not necessarily lead to a complete return to a physiological baseline. The more realistic goal would be a stable, robust state that can be sustained under long-term mechanical stress. This is consistent with the concept of robustness in a complex system, which may be maintained according to adaptive resilience rather than static balance (252).

Therefore, aortic dissection can be seen as a phase-transition-like biological process where nonlinear dynamics drive from compensated dysfunction to irreversible structural collapse. The potential therapeutic action should focus on the identification and modulation of critical destabilization states that prevent the system from crossing the critical point.

### 6.1 Redefining therapeutic logic: from end-stage repair to upstream network regulation

Current management of aortic dissection is typically reactive. Most of the patients are treated only after advanced structural failure occurs, and are treated in an event-driven manner with the focus on rapid hemodynamic repair and restoration of mechanical integrity. These treatments are life-saving but involve only late manifestations of disease and their potential sources of biological side-effects are limited and not beneficial for long-term disease progression or recurrence (253, 254).

Reframing aortic dissection as a vascular homeostasis imbalance disease requires a fundamental change of strategy, from end-stage structural correction to early modulation of upstream etiology network. Regulatory nodes such as vasa vasorum, hypoxia-induced metabolic reprogramming, oxidative stress, and inflammasome activation are key intervention targets, usually predating irreversible intimal degeneration and remaining reversible in early disease (255, 256).

Such downstream pathways may be beneficial in maintaining the function of the aortic wall. For instance, if one can simultaneously suppress pathological inflammatory signaling, restore mitochondrial function and redox balance, and stabilize smooth muscle cell phenotype then one could effectively limit matrix disruption and slow structural destabilization (257, 258).

Such upstream network control may however be highly controlled. Physiological processes including angiogenesis, immune monitoring, and metabolic stability are essential for homeostasis. Unregulated angiogenic or broad-spectrum anti-inflammatory therapy might interfere with the blood supply, delay repair or infection risk (259).The goal is therefore to resist, while dynamic modulation of the signal intensity would guide the system into a robust homeostatic state.

At a practical level, this highlights the need for biomarkers and imaging devices to detect metabolic and inflammation early, and for cell-specific and time-dependent drugs. Finally, integrating systems biology into clinical decision making may be useful for aortic dissection care to be more preventive and personalized.

### 6.2 Targeting adventitial dysfunction: stabilizing the outer layer of the vessel wall

Aortic blood vessels are composed of a vital microvascular network called the vasa vasorum that delivers oxygen and nutrients to the outer layers of the vessel walls. It is known that dysfunction of this system is a major component in early aortic diseases (260, 261). It should be stressed that increasing the number of microvessels does not guarantee adequate perfusion. In chronic inflammation, oxidative stress, and abnormal hemodynamics, newly formed vessels are structural and functional fragile, consisting of a closed basement membrane, a narrow endothelium, and poor junctional integrity (262).

Under pathological conditions, vascular endothelial growth factor (VEGF) is constantly upregulated, and blood vessel permeability increases. The compromised endothelial barrier allows plasma proteins, inflammatory mediators, and red blood cells to leak into the media (263, 264). Hemoglobin degradation releases heme and free iron, catalyzing oxidative stress via Fenton reactions, and the local oxidative-inflammatory processes are interleaved thus to link microvarian instability to medial degeneration (265).

Consequently, therapeutic strategies are shifting from pure anti-angiogenesis towards the concept of vascular normalization. The goal is not to reduce the number of vessels but to restore the structural integrity and functional capacity of the microvessels. The angiopoietin-1/2-Tie2 signaling axis plays a central regulatory role in this process. While Ang-1 maintains endothelial quiescence, stabilizes adherens junctions, and promotes pericyte recruitment, Ang-2, particularly in inflammatory environments, disrupts vascular architecture and increases permeability. Shifting this signaling balance toward Ang-1 dominance could strengthen VE-cadherin-mediated cell junctions, reduce leakage, and limit inflammatory cell infiltration (266, 267).

The aortic adventitia contains the vasa vasorum, an important microvascular system supplying the outer layers of the vessel wall with oxygen and nutrients. It is known that dysfunction of this system is a core mechanism in the early development of aortic disease (268). However, increased microvascular density does not necessarily lead to effective perfusion. In chronic inflammation, oxidative stress and abnormal hemodynamics, new vessels will often have immature structures and dysfunction, such as discontinuous basement membrane, insufficient endothelial coverage and poor junctional integrity.

Under pathological conditions, sustained upregulation of vascular endothelial growth factor (VEGF) drives angiogenesis and high permeability. The weakened endothelial barrier allows plasma proteins, inflammatory mediators and erythrocytes to leak back to the media. Hemoglobin breakdown releases heme and labile iron that catalyze oxidative stress via Fenton reactions, enhancing local oxidative and inflammatory cascades, which form a mechanistic link between microvascular instability and medial degeneration (269).

Besides the molecular effect, biomechanical factors such as vessel wall stress and abnormal strain patterns can cause inflammation of endothelial cells, block junction complexes, and even cause barrier dysfunction. Mechanic and biological factors could exert a combination of hemodynamic regulation (lower blood pressure, lower pulsatile load) and specific molecular treatment.

Other techniques, such as increasing pericyte-endothelial interactions, varying matrix structures for microvascular stability, and activating hypoxia signaling for aortic angiogenesis, are not fully evaluated yet and their clinical applications in aortics is at present under investigation. Overall, stabilizing the adventitial microenvironment is a potential upstream intervention to break the process of metabolic stress, inflammation, and structural collapse due to microvids dysfunction.

### 6.3 Targeting inflammatory amplification loops: limiting self-perpetuating tissue damage

The inflammatory response in aortic dissection not only has a secondary role, but also is highly self-amplifying and can drive and maintain tissue damage (270). Inflammatory cytokines, like IL-6 and TNF-α activate leukocyte recruitment, endothelial activation and vascular permeability, and also drive smooth muscle cells phenotypically to undergo phenotypic from a contractile to synthetic, inflammation-inducing, matrix-degrading phenotypes by upregulating proteases, such as MMP-2 and MMP-9, which lead to continuous matrix degradation and medial weakening (271, 272).

At the heart of this inflammation network is the NLRP3 inflammasome (NLRP3), which is a signaling mechanism linking metabolic stress to immune activation (273). Intracellular danger signals such as mitochondrial dysfunction, elevated reactive oxygen species, mitochondrial DNA release, ionic stress and accumulation of specific metabolic intermediates could activate the inflammasome (274). When assembled, the inflammasome triggers caspase-1, promote proliferation and secretion of IL-1β and IL-18, and pyroptosis, leading to temporary inflammatory signals as well as a long-term self-amplification, which further causes vascular damage (275).

These inflammatory loops are redundant and interconnected. Clinically, even after initial hemodynamic stabilization, a substantial proportion of patients exhibit sustained or recurrent systemic inflammation, which is independently associated with adverse long-term outcomes—suggesting that targeting a single pathway or a single time point may not provide durable suppression of the inflammatory process (276). The interest thus is focusing on strategies that can change not only the mediators, but also the upstream nodes or networks. For example, controlling inflammation activation, redox balance, and metabolism can affect multiple downstream pathways. Existing therapeutic approaches aim not only to completely suppress inflammation, but to restore the controlled inflammatory processes. While physiological inflammation is vital for host defense and tissue repair, the goal is to minimize the amplification without loss of reparative capacity (277, 278). This “threshold modulation” method seeks to keep the inflammatory signals within a range, such that healing is achieved rather than structural damage. To do so, it is time-and cell-dependent mediated interventions that are guided by biomarkers of inflammation.

At a translational level, since metabolic, oxidative, and inflammatory pathways are tightly coupled, combining drugs with strategies of metabolic and oxidative pathways can be beneficial (279). Clinical confirmation of these combinations in aortic dissection remains in its infancy, and is a topic for future research.

### 6.4 Modulation of metabolic reprogramming: restoring cellular energy adaptability

Metabolic reprogramming is a major adaptive response of vascular cells to microenvironmental damage in aortic dissection. Smooth muscle cells in diseased aortas often switch from mitochondrial oxidative phosphorylation to enhanced glycolysis, displaying a Warburg-like response like in proliferative and inflammatory cells (280). The metabolic shift can initially support cell survival under hypoxic conditions, but it results in pathological remodeling and functional decline.

Local lactate production is linked to increased glycolytic activity. Lactate is not just a metabolic product, it regulates gene expression and cell phenotype by triggering epigenetic changes such as histone lactylation and altering transcription programs for inflammation, matrix remodeling, and cell survival (281), while high lactate levels worsen the contractile role of smooth muscle and promote conversion to a synthetic phenotype and degrade the mechanical integrity of the aortic wall.

Metabolic reprogramming is correlated with mitochondrial dysfunction (282). With inadequate oxidative phosphorylation, ATP production is reduced, the electron leakage from the electron transport chain is more extensive and excessive ROS damage lipids, proteins and DNA, activate inflammatory signaling pathways and apoptosis processes in an unprecedented way (283, 284).

To minimize the damage done by oxidative phosphorous, targeting metabolic disturbances is highly promising. Restricting high glycolysis could remove the enzyme lactate dehydrogenase A and control glucose uptake via glucose transporter 1. On the same footing, protecting and restoring mitochondrial function would be important. *In-vitro* treatments such as mitochondrial targeted antioxidants, mitochondrial biogenesis and stabilizing electron transport chain’s function could help restore cell energy metabolism and oxidative stress (285).

Importantly, metabolic measures should consider cell-type specificity and time-dynamics. While inhibition of glycolyssis could reduce pathological inflammation, excessive suppression may be detrimental to essential functions such as cell survival and repair (286, 287). It should not be desired to fix the cells at one state, but to restore their robustness - the ability to adapt energy production paths with changing conditions.

### 6.5 Advances in extracellular matrix stabilization strategies: from terminal inhibition to network-level protection

Managing matrix integrity is a major problem in aortic dissection, and inhibition of matrix metalloproteinases (MMPs) is also a common strategy. However clinical results on direct MMP inhibition lack clear indication that matrix degradation is not a discrete pathology, but a downstream consequence of multilayer dysregulation involving inflammation, oxidative stress and fundamental cellular phenotype (288).

In the system, sustained inflammatory signalling and metabolic perturbations lead to MMP expression and activation. Inflammatory cytokines, reactive oxygen species and abnormal redox state inflammatory cytokines, reactive oxygen species and abnormal redox state converge to promote MMP expression and activation through both transcriptional and post-translational mechanisms (289, 290). In this way, effective matrix protection cannot be achieved with a monotherapy only on downstream networks.

Besides limiting matrix degradation, we are also interested in further increasing matrix synthesis and structural cross-linking. LOX and its isoforms promote collagen and elastin binding, which plays a key role in increasing the tensile strength and structural stability of the aorta (291). Higher LOX activity, or collagen maturation, could be complementary in stabilizing the extracellular matrix.

However, the biomechanical impact of matrix changes must be taken into account. Multidimensional collagen cross-linking can increase vessel wall stiffness, decrease compliance, impair the aorta’s elastic storage capacity and increase hemodynamic stress. Because the aorta is a delicate balance between strength and elasticity, one would like to retain both resistance to rupture and the capacity to buffer pulsatile blood flow (292, 293). This reflects the fact that the matrix is not a passive structure but a living active part of a dynamic mechanobiological system. Future work will focus on coordinated regulation of matrix metabolism, cell phenotype, and mechanical stress that achieve network-level protection, not isolated inhibition. .

The central pathological network comprises three interconnected nodes forming a self-amplifying loop: metabolic reprogramming (glycolysis, succinate, ROS), NLRP3 inflammasome activation (IL-1β, IL-6, TNF-α), and ECM degradation (MMP-2/9, elastin fragmentation). Vasa vasorum dysfunction (microhemorrhage, iron release, HIF-1α stabilization) serves as the upstream driver. Four therapeutic modules target distinct levels: iron chelation and ferroptosis inhibition to block oxidative stress; NLRP3 and cytokine inhibitors to suppress inflammatory amplification; metabolic modulators (LDHA inhibition, mitochondrial protection) to restore metabolic flexibility; LOX agonists, MMP inhibitors, and vasa vasorum normalization to stabilize ECM and microvasculature. The overarching goal is to restore vascular homeostasis through coordinated multi-level network modulation rather than single-target blockade. Abbreviations: DAMP, damage-associated molecular pattern; ECM, extracellular matrix; HIF-1α, hypoxia-inducible factor 1α; IL, interleukin; LDHA, lactate dehydrogenase A; LOX, lysyl oxidase; MMP, matrix metalloproteinase; NLRP3, NLR family pyrin domain containing 3; ROS, reactive oxygen species; TIMP, tissue inhibitor of metalloproteinases; TNF-α, tumor necrosis factor α; VV, vasa vasorum.

### 6.6 Transforming the clinical research paradigm: towards process-oriented precision medicine

**Figure 7:**
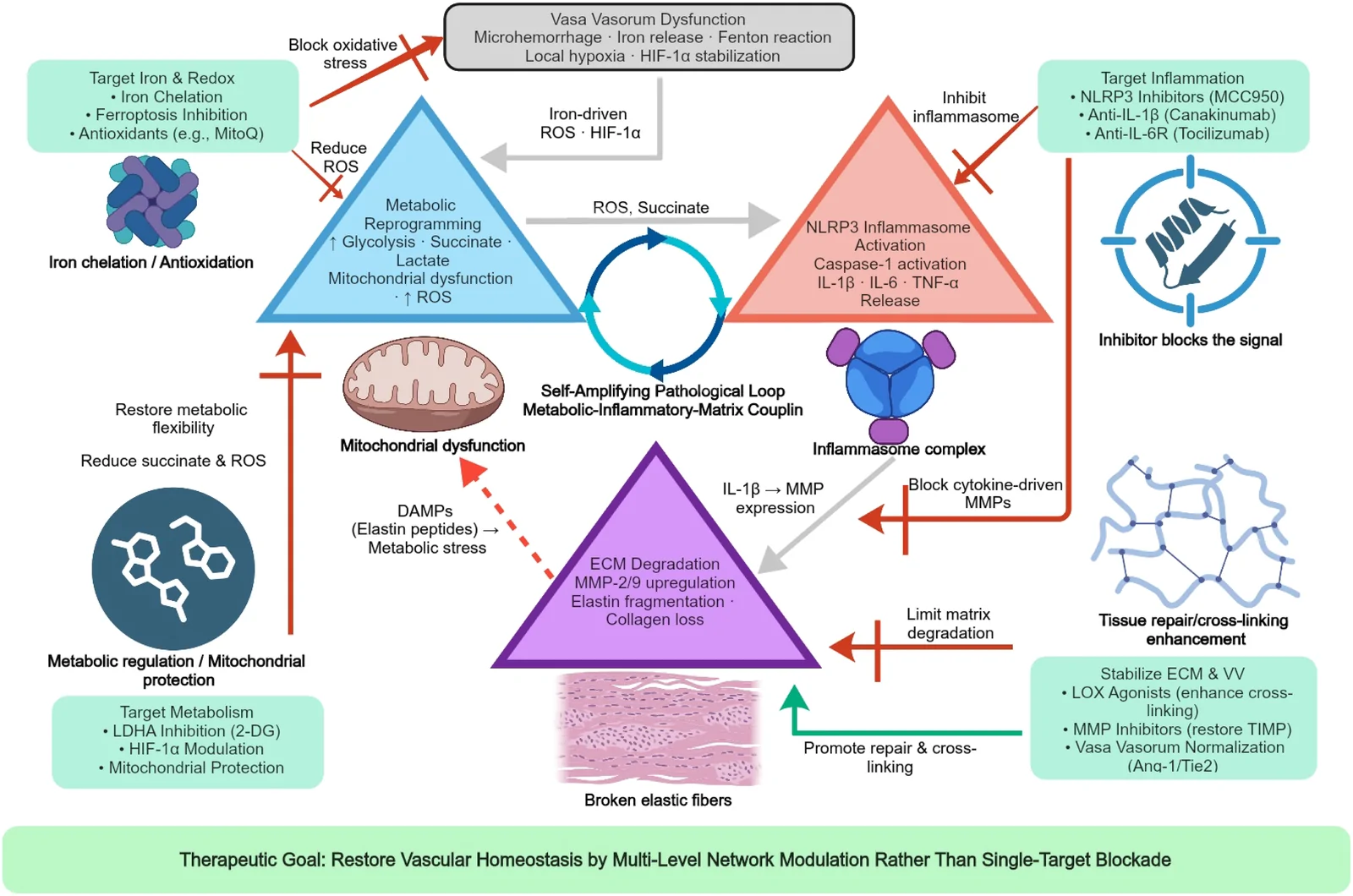
Systems-level therapeutic targeting of inflammation–metabolism–ECM coupling in aortic dissection.

Recent advancements in imaging and multi-omics models facilitate aortic dissection research from event-based endpoints to process-oriented models focusing on disease (**Figure 7**). Traditional clinical models are focused on detection and management of overt dissections, yet more research is currently finding that important pathological processes occur long before structural failure causes clinical symptoms (294, 295).

High-resolution magnetic resonance images such as vessel wall-specific and perfusion-sensitive sequences allows measurement of the concentrations of vasa vasorum, permeability and local tissues. Fluorodeoxyglucose PET/CT represents metabolic activity and inflammation in the aorta wall (296, 297). Changes in these imaging parameters often precede anatomical abnormalities and thus can be used for early risk stratification and intervention. Multi-omics analyses combining genomics, transcriptomics, proteomics, and metabolomics can also characterize disease heterogeneity at the individual level, finding molecular signatures of inflammation, metabolic reprogramming, matrix remodeling and cellular stress. These tools can be combined with machine learning, network analysis, which can reveal patterns that might predict instability or rapid growth.

Moreover, imaging and molecular profiling provide a way to develop personalized risk models beyond traditional measures of aortic diameter and blood pressure. Risk stratification could take into account dynamic biological states such as metabolic activity, inflammatory intensity and microvascular integrity along with anatomical features (298). This transition towards biological phenotyping is a major step forward for precision medicine in aortic disease. If realized in clinical practice, it could fundamentally change management by reducing reactive interventions after dissection to active homeostatic modulation of the asymptomatic state, leading to reduction of acute catastrophic events and consequently to long-term disease change that greatly alters the natural history of aortopathies.

There is still room for improvements including standardizing imaging protocols, incorporating multi-omics data into clinically convenient settings, and testing in large prospective cohorts. These challenges cannot be overcome to realize process-centered precision medicine in aortic diseases.

As summarized in **Table 5**, the future treatment logic is shifting from end-stage structural repair to early intervention of the upstream regulatory network. By targeting the trophic vascular axis, inflammatory axis, metabolic axis, and structural axis respectively, we expect to achieve a paradigm shift from “repairing damaged structures” to “restoring system homeostasis”. Each intervention strategy aims to correct the imbalance of homeostasis at a specific level, ultimately jointly enhancing the overall robustness of the aortic wall and preventing it from crossing the critical point. However, to translate these promising strategies into clinical practice, we still need to address the numerous challenges and controversies existing in current research.

**Table 5:** Therapeutic strategies targeting key nodes of the homeostatic network: a multi-axis intervention framework.

Targeted axis	Key molecular target	Intervention method example	Homeostatic restoration mechanism
Vasa vasorum axis	VEGF/Ang2	VEGF Antagonist/Ang1 Analog	Stabilizes microvascular barrier, reduces microhemorrhage and leakage
Inflammatory axis	NLRP3/IL-6	MCC950/Tocilizumab	Blocks inflammatory amplification, inhibits MMPs-mediated degradation
Metabolic axis	LDHA/Mitochondrial ROS	2-DG/MitoQ	Correct glycolytic shift, restore VSMC contractile phenotype
Structural axis	LOX/MMP-2,9	LOX agonists/Combined inhibition strategy	Enhance elastic fiber cross-linking, improve mechanical resilience

## 7 Challenges, controversies, and future perspectives

Though multilayer homeostasis imbalance and mechanistic information of endothelial dysfunction, inflammatory activation, and metabolic reprogramming can provide a general systemic explanation of the origin of aortic dissection, there is still a great gap between mechanistic understanding and clinically-predictable tools (299, 300). While current research reveals that aortic wall becomes structurally unstable, it is still very difficult to predict when an acute dissection will occur or at what threshold tissue damage becomes irreversible.

This gap highlights a key limitation of the research: the mechanistic studies have not yet yielded a meaningful improvement in prediction ability. Clinically, this means that while an increasing amount of scientific information is becoming available about biological processes, we still do not identify high risk individuals before a catastrophe occurs (301). Thus, one of the main challenges for aorta dissection research may not be the description of end-stage structural failure, but the description in general of the often brief, asymptomatic transition from early molecular dysregulation to catastrophic mechanical failure.

From a systems biology point of view, such a transition is likely to be a critical transition characterized by nonlinear dynamics, threshold effects, and increased susceptibility to perturbations; multidimensional changes - smooth muscle cell phenotype switching, metabolic adaptation, cumulative oxidative stress, and microvascular remodeling are involved that progressively weaken structural robustness (302, 303). Unlike linear disease models, this process is closely related to a critical transition in complex systems where slow changes can suddenly lead to irreversible effects.

At the moment, no reliable biomarkers or imaging results can capture this pre-dissection “critical window” before a rupture and reflect dynamical biological states instead of static structural features, and can incorporate information at molecular, cellular, and tissue levels. Longitudinal imaging, circulating biomarkers and numerical modelling can address this problem, and they are not yet being used clinically.

### 7.1 Limitations of current models

The available mechanistic models that give a good account of the causes of the disease however lack supporting evidence. The main issue is that there is little longitudinal evidence to support the causal explanation. Most evidence comes from cross-sectional analysis of surgically resected aortic tissue or end-stage disease samples. Even if these can give molecular signatures of advanced disease stages – upregulation of inflammatory mediators, matrix-degrading enzymes, hypoxia, etc., they cannot fully describe the time scale of disease onset and progression. For example, molecular abnormalities associated with NLRP3 inflammasome elements, MMP-9 and HIF-1α may be well known but it is unclear whether they are the cause of the onset of disease or secondary responses to known structural damage (304–306). Refuting causality from fixed endpoint data is unpredictable and results in oversimplification of the correlative findings.

Another major problem of our findings is a lack of sample size and heterogeneity of available clinical data. Clinical studies on aortic dissection are currently only single-center small-cohort studies that do not capture all disease phenotypes. The dissection population is highly heterogeneous; some patients are driven by chronic hypertension and biomechanical stress, while others may be driven by inflammatory activation or metabolic abnormalities (307, 308). Individual differences in the relative contributions of various causes of an individual may cause inconsistent study results and make conclusions difficult to be made. Without large multicenter, prospective cohort studies, it is still a challenge to test and test new theoretical models. Without these sets, it remains elusive to find reliable risk stratification systems or explain how different causes of different processes interact with time in an individual.

Methodologically, these limitations suggest that research needs to shift from static description to dynamic tracking. Future studies should prioritize reconstructing the temporal progression of the disease through longitudinal imaging, serial biomarker monitoring, and multi-omics analyses. Only by dynamically capturing the temporal interactions between cell states, metabolic networks, and biomechanical factors can mechanistic models achieve true causal explanatory power and clinical utility.

### 7.2 Major technological challenges and future perspectives

#### 7.2.1 Non-invasive quantitative imaging of the vasa vasorum

While these aortic wall microvascular findings are important, it is still not clear how the vasa vasorum can function *in vivo*. Current evidence is mainly from pathohistological observations that provide high spatial information but they only provide ex vivo and static endpoint information. Modern clinical imaging approaches like CT or MRI report macroscopic structural changes, but do not provide functional information like microvascular perfusion, permeability or local oxygenation (309).

This raises a major issue with the current diagnosis: current imaging tools are the outcome of the disease, rather than biological processes. In order to fill this gap, increasing efforts are being made towards molecular and functional imaging to observe microvascular processes *in vivo*, e. g., specific probes for angiogenesis and hypoxia markers like VEGFR2 and intra-alpha-alphavβ3 and hypo-channel pathways, as well as high-resolution MRI, dynamic perfusion imaging, and PET (310, 311). They would enable us to estimate microvascular density, permeability, inflammatory activity, and tissue oxygenation simultaneously and identify focal “high-risk areas” in the aortic wall before major structural damage can occur.

There are several challenges before clinical use: the limited spatial resolution of microvascular images, variation in signal specificity and the need for robust validation from large prospective cohorts. Safety of contrast agents and radiotracers must be carefully checked, imaging protocols and quantitative measurements need to be standardized in order to be reproducible and comparable across sites (312, 313).

#### 7.2.2 Development of therapeutic strategies to restore vascular homeostasis

From a system biology perspective, if aortic collapse follows progressive loss of vascular homeostasis, then relying solely on single molecular pathways is unlikely to be effective. Biological networks of inflammation, metabolism and vascular remodeling are both highly redundant and compensated: intervention at one node is often counterbalanced by parallel pathways (314).

In addition, we are increasingly interested in drugs which are not only designed to suppress individual effector molecules but to stabilize the network, where it is particularly promising to target key nodes of the metabolic-inflammatory axis. Such molecules are important in signaling cellular stress and energy homeostasis including mitochondrial reactive oxygen species in mitochondria, AMPK signaling, glycolytic flux (315, 316).

Modulating these pathways can decrease inflammation, enhance immune function and preserve smooth muscle cell function. However, clinical translation issues are inherent. Metabolization programs in vascular cells are highly relevant to important physiological functions like tissue repair, proliferation and immune defense; inadequate or inaccurate treatment would cause confounding or off-target effects.

Future drug development should focus on context-dependent, cell-specific modulation options, such as delivery systems, or time-varying approaches. Long-term safety and effectiveness should be tested in clinical situations before entering the standard of care.

#### 7.2.3 Development of integrative prediction models

Current clinical risk stratification for aortic dissection is based on simple parameters such as the aortic diameter and blood pressure. These are clinically relevant but may not explain the intrinsic biological instability (317, 318). As discussed in this review, certain pathological processes, like inflammation, metabolism, and microvascular dysfunction can occur prior to overt anatomical changes.

Future prediction models need to incorporate multi-dimensional data of imaging features, circulating biomarkers, genetic variants and metabolic profiles to capture fully the biological range of the disease. Such models could also identify high risk individuals in the early stages of the progression of the illness and guide treatment. AI and machine learning provide powerful tools to analyze high-dimensional information and search for possible patterns not found in statistics. Imaging markers, molecular data, and clinical data are allowed to integrate these features to extract association risk signatures of impending structural instability.

Balancing between prediction performance and interpretability: Although complex models provide high accuracy, they are not transparent which reduces clinical trust. Genetic data have been recently integrated into risk models with successful validation across different populations—for example, a polygenic risk score based on a large biobank was able to predict thoracic aortic dissection, laying the groundwork for genotype-based stratification (319). Predictive tools need to move past risk estimation toward clinically relevant mechanistic insight; future efforts will focus on expanding these genetic models incorporating additional biological aspects—including imaging features and circulating biomarkers—in an interpretable framework and validating them prospectively in multicenter cohorts so as to improve generalizability and real-world clinical utility.

### 7.3 Future research strategies

Due to these problems, future aortic dissection studies must be able to develop a holistic multiscale model between experimental biology, clinical studies, and technological innovations. Only then can current mechanistic insights translate into predictive and actionable clinical practice.

At the same time, experimental models are essential in understanding disease mechanisms, but the model should be optimized to better capture the dynamics of aortic disorders. Typical single timepoint analyses present only static snapshots and can hardly be used to recover the temporal history of pathological events (320). Longitudinal experimental designs with multi timepoint sampling should be used in order to characterize inflammatory signaling, metabolic reprogramming, matrix remodeling and biomechanics in disease stages and determine the temporal ordering of molecular and cell events causing the collapse. Lineage tracing, single cell technologies, and spatial multi-omics may also help to understand the temporal dynamics of cell state transitions and cell-cell interactions at high resolution (321).

In clinical settings, large future multicenter cohort studies will help to understand the history and heterogeneity of aortic dissection. Serial longitudinal followups in high-risk populations involving sequential imaging, circulating biomarker profiles, and clinical phenotype may help to identify key transitions from mild disease to overt disease (322, 323). These datasets will not only validate and fine-tune existing mechanistic models, but will also lead to personalized risk prediction models based on dynamic biological states, rather than fixed anatomical thresholds.

Technological breakthroughs will play a crucial role in this integration. Recent advances in combining high-resolution imaging and multi-omics data allow both spatial and molecular features of the aortic wall to be described simultaneously. For example, joint comparison of spatial transcriptomics and imaging features can be used to determine relationships between focal inflammatory activity, metabolic stress, microvascular disorders and structurally vulnerable areas (324, 325). Establishing standardized workflows to integrate such heterogeneous types of data will be necessary for translating research findings to reproducible scientific and clinical tools.

Developing treatments in a stepwise pathway to clinical applications must be done stepwise. First, an initial study will assess the safety and effectiveness of interventions to homeostasis, such as control of the metabolic-inflammatory coupling or targeted delivery systems, before going to large controlled clinical trials that determine disease progression, stability and clinical outcomes. Including biomarker and imaging endpoints into a trial design may give more sensitive measures of treatment effects than clinical events alone.

In general, future research should rely on a closed-loop model that tightly integrates discovery, validation, and application. Experimental models inform clinical studies, and observations of patients continuously refine mechanistic analyses, and such a bi-directional integration can help to bridge the gap between current mechanistic information and prediction.

Overall, aortic dissection is moving towards a predictive and preventive medicine. Time resolution, spatial detail and data integration will be essential in spotting early signals of system instability before collapse in an effective manner, lead to true early intervention, change of natural disease path, and reduce the burden of acute catastrophe.

**Table 6** summarizes these research strategies, which form a closed-loop path from the laboratory bench to the bedside, and then back to the mechanism study. They collectively point to a core goal: to change the current situation in the field of aortic dissection where “passive response to catastrophic events” prevails, and shift to a new paradigm of “active monitoring and regulation of biological risks”. Only through this multi-dimensional, interdisciplinary, and integrative effort can we hope to transform the decades of mechanism research results into clinical tools that can effectively reduce the incidence and mortality of aortic dissection.

**Table 6:** Future research roadmap: integrating deep validation, clinical cohorts, and technological innovation.

Research dimensions	Core recommendation strategy	Expected scientific outputs
Deep experimental validation	Combine spatial transcriptomics with conditional gene knockout animal models to conduct multi-time-point “full-disease-course” tracking.	Elucidate the precise molecular switches and temporal logic of the inflammatory storm triggered by VV leakage.
Prospective clinical cohort	Establish a large-scale prospective biospecimen and imaging repository covering patients with hypertension and hereditary aortic diseases.	Identify highly sensitive early warning biomarkers and validate homeostasis imbalance thresholds.
Integrated technology development	Develop an “imaging-omics” integrated model utilizing AI to extract characteristic electrical or imaging signals indicative of microscopic vascular wall damage.	Achieve an automated assessment system transitioning from “structural monitoring” to “biological risk prediction. “
Innovative intervention trials	Commencing with small-sample trials of drug repurposing (e. g., metformin for metabolic regulation), progressively expanding to Phase II/III clinical trials targeting VV stabilization.	Validate the clinical value of “steady-state restoration” therapies in reducing dissection incidence and postoperative recurrence rates.

## 8 Conclusion

Although the usual definition of aortic dissection is a serious structural injury to the aortic wall, there is increasing evidence that it is actually a long-term and multi-scale degradation of the internal homeostasis of the arteries (326, 327). We propose a global framework in which, while vascular dysregulation, hypoxia-induced metabolic reprogramming and improved oxidative stress-induced inflammation are not isolated phenomena, they are an essential part of a highly coupled dynamic self-reinforcing system in which cell phenotype, metabolic state, matrix quality and biomechanical effects are interacting. Early small disruptions may be compensated, and the system can still be seen as structurally stable under certain conditions on the surface, but beyond a certain threshold nonlinear amplification may quickly accelerate to irreversible tissue damage, resulting in intimal rupture and aortic severities. This unites seemingly contradictory clinical characteristics of sudden onset and long-term precursors. It has dramatically changed the field of research and clinical practice - from late structural repair to early disease detection and control of upstream pathogenic processes. Its main point is that there is a short asymptomatic time step, in which intervention before physiological structural changes may be reversible. What to look for and how to track and control the dynamics of the system is a major challenge and opportunities.

**Figure 8:**
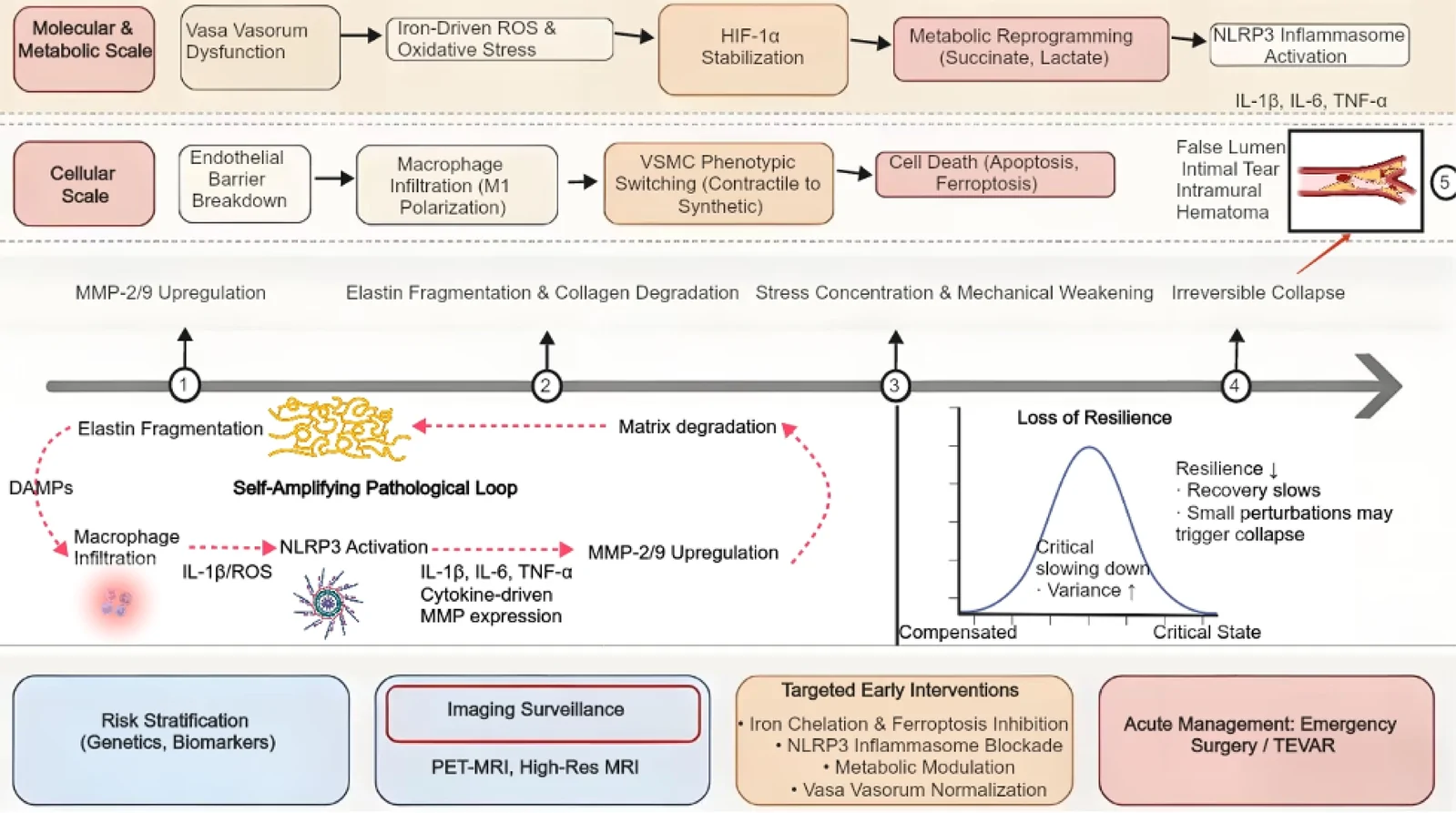
Multiscale dynamic model of aortic dissection: from homeostatic imbalance to critical transition.

In summary, **Figure 8** summarizes the core theoretical model proposed in this paper. It depicts how the aortic wall, starting from a highly robust steady-state system, undergoes a multi-scale, gradual destabilization process, eventually crossing the critical point and erupting into a catastrophic dissection event. This dynamic model emphasizes several key transitions: from a period dominated by negative feedback for compensatory adaptation, to a period where positive feedback loops prevail; from local, reversible molecular events to systemic, irreversible structural collapse. It suggests that the “golden window” for clinical intervention may not lie in dealing with the already formed dissection, but in identifying and regulating that long and hidden “pre-critical state”. To achieve this goal, we need to combine molecular imaging, circulating markers, and computational biology to develop new tools that can dynamically assess the “systemic homeostatic reserve” of the aortic wall, thus truly opening a new chapter in the preventive medicine of aortic diseases.
